# Naked-Eye Thiol Analyte Detection via Self-Propagating, Amplified Reaction Cycle

**DOI:** 10.1021/jacs.3c02937

**Published:** 2023-09-25

**Authors:** Benjamin Klemm, Ardeshir Roshanasan, Irene Piergentili, Jan H. van Esch, Rienk Eelkema

**Affiliations:** Department of Chemical Engineering, Delft University of Technology, Van der Maasweg 9, 2629 HZ Delft, The Netherlands

## Abstract

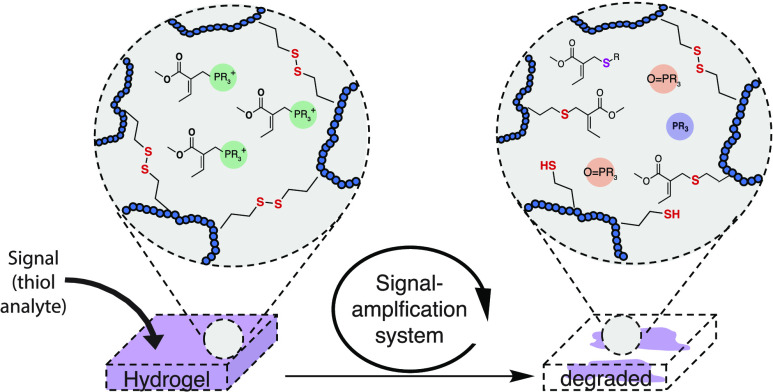

We present an approach for detecting thiol analytes through a self-propagating amplification cycle that triggers the macroscopic degradation of a hydrogel scaffold. The amplification system consists of an allylic phosphonium salt that upon reaction with the thiol analyte releases a phosphine, which reduces a disulfide to form two thiols, closing the cycle and ultimately resulting in exponential amplification of the thiol input. When integrated in a disulfide cross-linked hydrogel, the amplification process leads to physical degradation of the hydrogel in response to thiol analytes. We developed a numerical model to predict the behavior of the amplification cycle in response to varying concentrations of thiol triggers and validated it with experimental data. Using this system, we were able to detect multiple thiol analytes, including a small molecule probe, glutathione, DNA, and a protein, at concentrations ranging from 132 to 0.132 μM. In addition, we discovered that the self-propagating amplification cycle could be initiated by force-generated molecular scission, enabling damage-triggered hydrogel destruction.

## Introduction

Living systems are commonly able to quantitatively detect and process (bio)chemical signals.^1−3^ In contrast, synthetic analogues capable of detecting and amplifying an external signal input using only chemical reactions, and not requiring enzymatic transformations, are exceedingly rare and only a few systems^2,4−9^ exist. Signal-responsive materials capable of translating and amplifying a signal^10^ into a global macroscopic change will find many applications ranging from biomedical sensors,^11,12^ advanced forensics^13^ to socioenvironmental diagnostics^13−16^ (assays to detect, e.g., food pollutants, explosives, or disease markers). Traditionally, chemosensors allow for sensitive detection by amplification of a reporter signal via a signal detection event, which is frequently coupled with photoluminescence or colorimetry to obtain an optical read-out.^3^ A variety of chemical signals have been used as active triggers to initiate the self-propagating amplification reaction on reagents, including hydrogen peroxide,^7^ thiols,^6,17^ and fluoride,^5,18−21^ among others.^4,8,22^

So far, studies on signal-amplified responsive synthetic materials using small molecule reagents^23,24^ or self-immolative polymers^25−27^ have been limited due to their challenging synthetic procedures to access reagents or polymers and issues with background interference.^3,10,28^ An alternative approach to achieve bio-inspired amplified response in synthetic soft materials is the β-mercaptoethanol (BME) or dithiothreitol (DTT)-mediated amplification cascade based on Meldrum’s acid-conjugated polymeric materials.^15,28^ This strategy, first described by Anslyn and co-workers,^9^ detects thiol signals indirectly via the thiol-exchange-mediated release of BME and its subsequent reaction with a Meldrum’s acid-based reagent. Once initiated, the reaction converts one equivalent of BME or DTT to decouple two equivalents of thiol. Using this approach, the authors were able to amplify the initial thiol input and convert it to macroscopic material degradation and optical detection.^15^ Inspired by these concepts, we sought to develop a new strategy for creating soft polymeric materials that are able to recognize, amplify, and translate (bio)chemically relevant signals into global macroscopic material changes, regardless of the quantities or molecular size of the applied signal. To realize this, we developed a new molecular approach for signal amplification in aqueous buffer by using allylic substitution of electron-deficient allyl acetates with trivalent phosphines as signal amplifiers. More commonly, electron-deficient allyl acetates are used together with tertiary nitrogen nucleophiles^29−31^ to form positively charged Morita–Baylis–Hillman acetate adducts. In contrast, the developed amplifier is based on an inactive phosphorous moiety and an electrophilic double bond, which enables nucleophile-triggered substitution, converting the amplifier back into a neutral phosphine species. To the best of our knowledge, the controlled release of trivalent phosphines from allylic phosphonium salts^32−35^ has not been shown so far.

This strategy enabled (i) control over disulfide redox chemistry by using the signal-responsive allylic phosphonium ion, capable of direct thiol analyte recognition and corresponding release of a phosphine species; (ii) coupling of this chemistry to a macroscopic response of a cross-linked hydrogel. Upon signal detection, the gels undergo physical degradation through chemical cascade reactions within the gel matrix. We were able to detect multiple analytes across a wide concentration range and realized mechanical cascade initiation by cutting the gels, demonstrating damage-triggered material response.

## Results and Discussion

**Figure 1:**
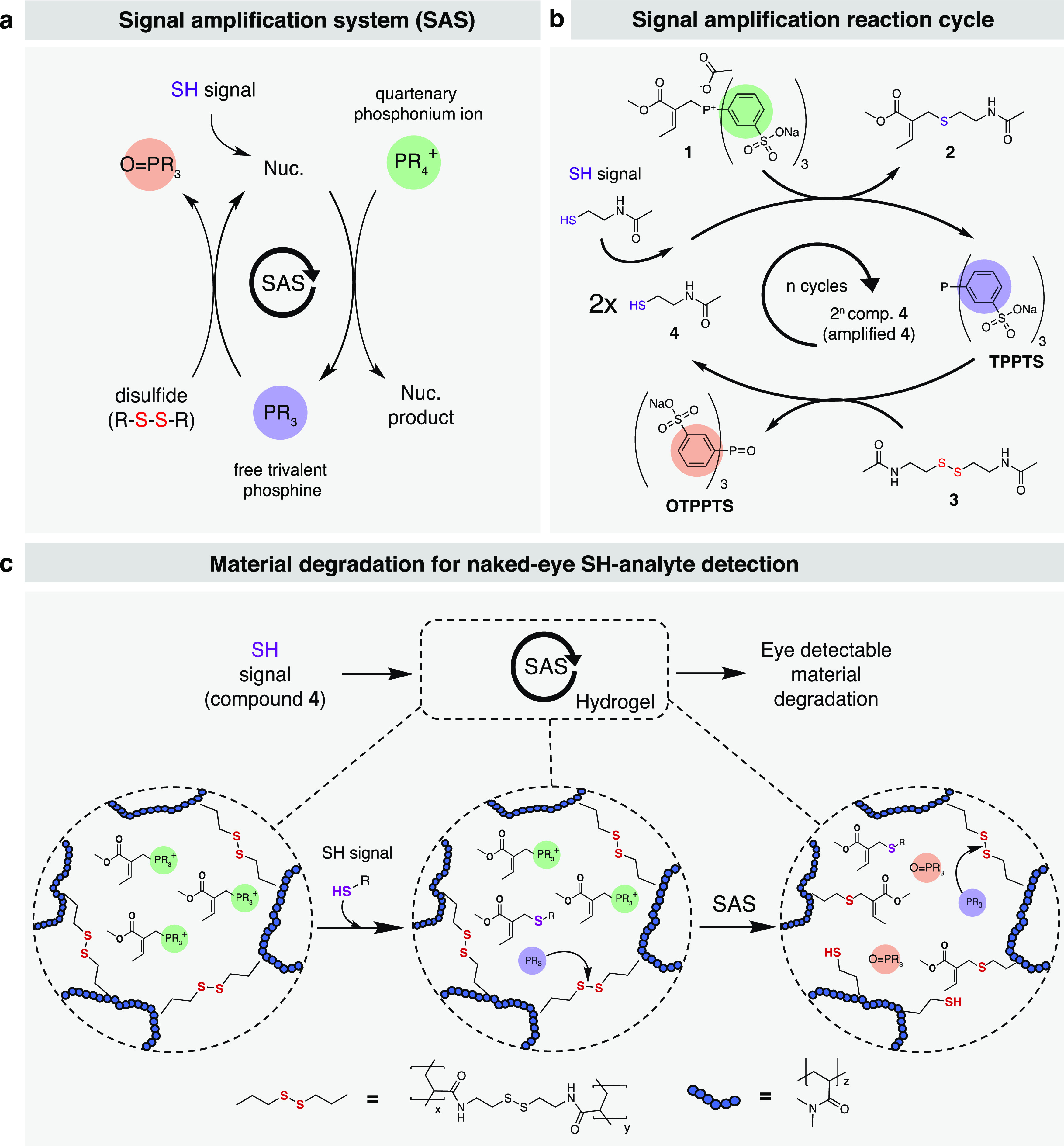
Schematics of the signal amplification system (SAS), its conditions, components, and material degradation mechanism. (a) Generic SAS, consisting of nucleophilic substitution and disulfide reduction reaction. (b) Chemical structure of allylic phosphonium salt **1**, substitution product **2**, free phosphine (TPPTS), disulfide **3**, thiol **4**, and oxidized phosphine (OTPPTS). Specifically, TPPTS is liberated from **1** upon SH-signal (compound **4**), which initiates disulfide reduction. The reduction reaction produces additional thiols, which themselves continue to liberate more TPPTS. This cascade results in an amplification of the starting thiol signal. (c) BAC cross-linked DMA hydrogels used for naked-eye SH-analyte detection. Upon signal addition, the liberated TPPTS inside the hydrogel matrix reduces disulfide cross-links, which themselves liberate more TPPTS. This results into a signal-triggered self-propagating amplification and ultimately into the degradation of the hydrogel material.

The signal-triggered amplification system (SAS) consists of two reactions in aqueous buffer (Figure 1). First, we activate a phosphine (PR_3_) compound by allylic substitution on a quaternary allylphosphonium ion (PR_4_^+^) with thiol nucleophiles (SH-signal), which forms an allylic reaction product (nuc. product). In the second reaction, the liberated trivalent phosphine reduces a disulfide bond resulting in the formation of two thiol equivalents and the production of phosphine oxide (O = PR_3_) (Figure 1a). The two new thiol molecules can perform another allylic substitution reaction on the remaining allylphosphonium salt, leading to the liberation of more phosphine and subsequent disulfide reduction, propagating the cycle. As this iterative process continues, the quantity of thiol molecules is amplified, until all neutral phosphine is consumed.

In this new molecular approach for signal amplification, we employ allylic phosphonium salt **1**. We were able to liberate trisodium tris(3-sulfophenyl)phosphine (TPPTS) from **1** upon the addition of highly nucleophilic thiols^36,37^ such as N-acetyl-cysteamine **4** (Figure 1b). To ultimately achieve thiol amplification, we further evaluated disulfide reduction by liberated TPPTS using N,N′-diacetylcystamine **3**. Using optimized conditions, substoichiometric amounts of **4** are able to initiate the system, resulting in the formation of byproduct **2** and the release of TPPTS. The liberated TPPTS then reduces **3**, forming two equivalents of **4** and one equivalent of phosphine oxide (OTPPTS). The equivalents of thiol formed in *n* cycles will theoretically be equal to 2^n^. Two thiols initiate the release of two additional equivalents of TPPTS, and consequently the process is self-propagating leading to an amplification of **4** (Figure 1b).

Having established the amplification system, we then sought to apply this chemistry to a synthetic material. For this, we fabricated hydrogel structures using *N*,*N*-dimethylacrylamide (DMA) with *N*,*N*′-cystamine bis(acrylamide) (BAC) cross-links (3.5 wt %) by free-radical polymerization.^38,39^ We anticipated that upon SH-analyte sensing, compound **1** releases TPPTS inside the redox active material matrix. Subsequent amplification reactions reduce internal disulfide cross-links, thereby physically transforming the hydrogel from gel to sol, making the process visible to the naked eye (Figure 1c). Since analyte detection occurs through the amplification system, only substoichiometric amounts of SH-analytes are needed to trigger material dissolution. Consequently, we studied the material at hand by exposing it to various biologically relevant SH-analytes and evaluated their sensitivity and application for naked-eye analyte detection.

### Kinetic Control over Phosphine Activation

**Figure 2:**
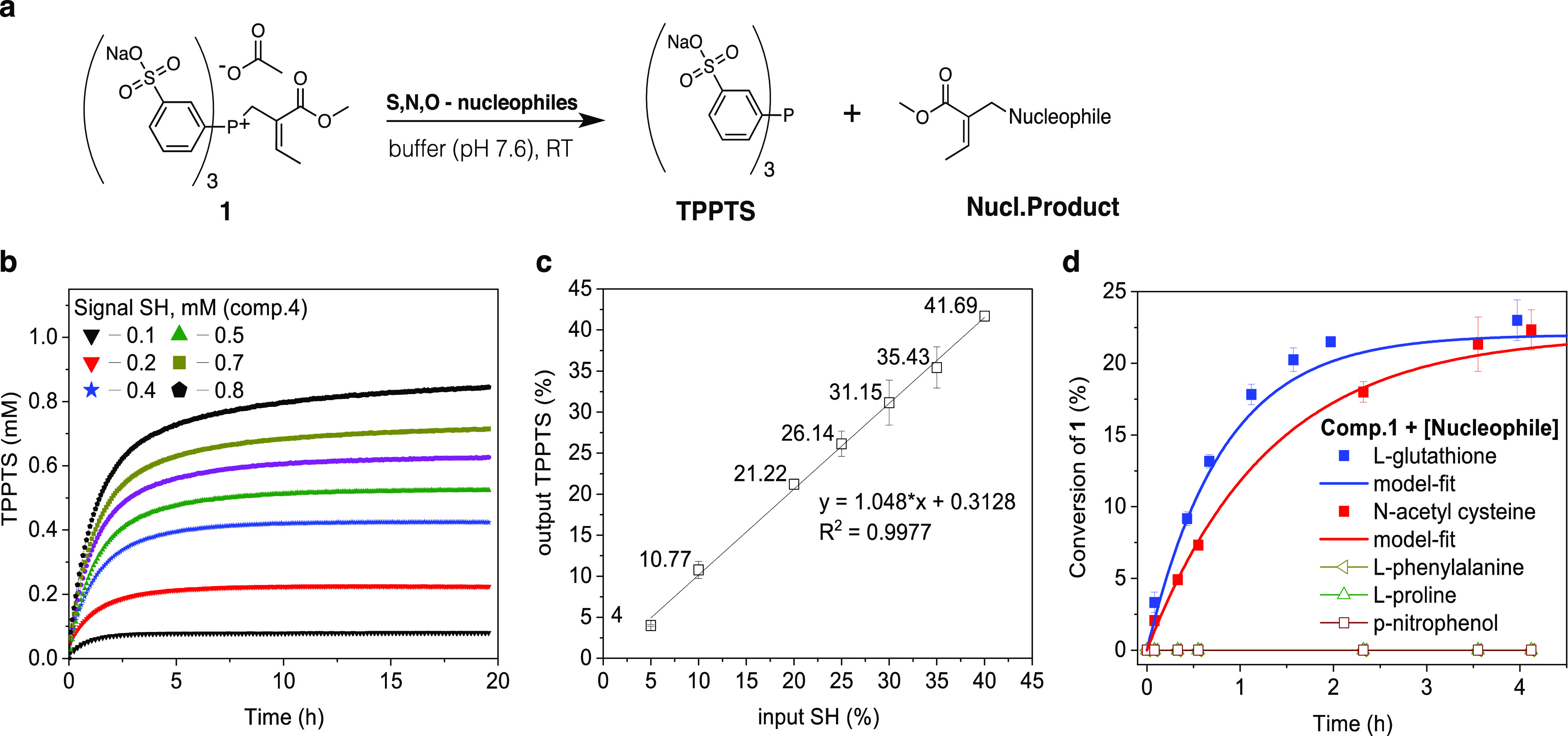
Kinetic control over TPPTS release. (a) Nucleophilic substitution reaction of compound **1** with S-, N-, or O-terminal nucleophiles (l-glutathione, N-acetyl cysteine, l-proline, l-phenylalanine, and p-nitrophenol), forming the nucleophilic substitution product and releasing TPPTS. (b) Reaction of compound **1** with a range of **4** (SH-signal) concentrations**,** forming 2-(acetylamino)ethanethiol (**2**) and TPPTS. TPPTS concentration vs time for a range of **4** concentrations. (c) SH-signal input (%) and TPPTS output (%) diagram, showing the control of TPPTS release upon the addition of **4**. Reactions were monitored by UV–vis at 260 nm and performed in duplicate. Conditions: phosphate buffer (0.1 M, pH 7.6), RT, 20 h, 2 mM of **1** and indicated amounts of **4**. (d) NMR reactivity study using 0.067 mM of **1** (1.0 equiv) and 0.20 equiv of a range of different nucleophiles (indicated in the figure) in aqueous buffer (2:8 D_2_O: phosphate buffer (0.1 M, pH = 7.6)) at 25 °C. Error bars represent standard deviation from duplicate runs. Solid lines represent the k-value model fit to the experimental data.

To evaluate the efficacy of the amplification strategy, we studied the SH-triggered release of TPPTS from compound **1**. Nucleophilic substitution with S-terminal nucleophiles on **1** results in the release of TPPTS and the formation of the nucleophile product (Figure 2a). We first exposed 2.0 mM of **1** to substoichiometric amounts of **4** ranging from 0.1 to 0.8 mM and monitored the release of TPPTS by UV–vis absorbance at 260 nm (Figure 2b). When 40% (0.8 mM) of thiol was used (vs. compound **1**), we observed a complete release of TPPTS within ∼20 h (Figure 2b). As expected, the release was slower when less thiol was added to the system. However, the release of TPPTS could stoichiometrically be correlated to the amount of SH input. Indeed, we found a linear correlation (*R*^2^ = 0.99) between thiol input and released TPPTS (Figure 2c).

In addition, we conducted a reaction rate study to further understand the reactivity of **1** toward S-, N-, and O-terminal nucleophiles, with their order being l-glutathione > N-acetyl cysteine (Figure 2d and Supporting Figures 6 and 7). We can attribute the kinetic variations (*k*_GSH_ = 240 ± 14.8 vs *k*_cysteine_ = 142 ± 2.7 M^–1^ h^–1^) between different thiol compounds to the difference in nucleophilicity of the employed S-terminal nucleophile. In contrast, N-terminal nucleophiles, including l-proline and l-phenylalanine, did not react with **1** (Supporting Figures 8 and 9). A similar observation was made for p-nitrophenol (Supporting Figure 10). This shows that the system displays a high sensitivity (Figure 2b) and selectivity (Figure 2d) toward S-terminal nucleophiles. Importantly, we did not observe the interference of the Michael addition or other side reactions during our experiments.

As control, we tested compound **1** without **4** in phosphate buffer (0.1 M, pH = 7.6) as well as in cell culture media (DMEM) by monitoring it over ∼24 h with ^1^H NMR spectroscopy (Supporting Figures 2 and 3). Over this time, we found that **1** is stable in the absence of SH-signal (in buffer) as we did not observe the release of TPPTS via hydrolysis. Remarkably, even in cell culture media, containing many amino acids, no system initiation was observed (note: DMEM also contains 0.2 mM of cystine HCl, the oxidized (dimerized) form of cysteine). Background interference such as hydrolysis or side reactions without trigger has been a consistent issue among self-propagating amplification reactions.^10^ TPPTS alone in aqueous media showed negligible oxidation, forming less than 5% OTPPTS during the 24 h experimental period under air (Supporting Figure 4).

### Kinetic Modeling of Phosphine Activation and Disulfide Reduction

**Figure 3:**
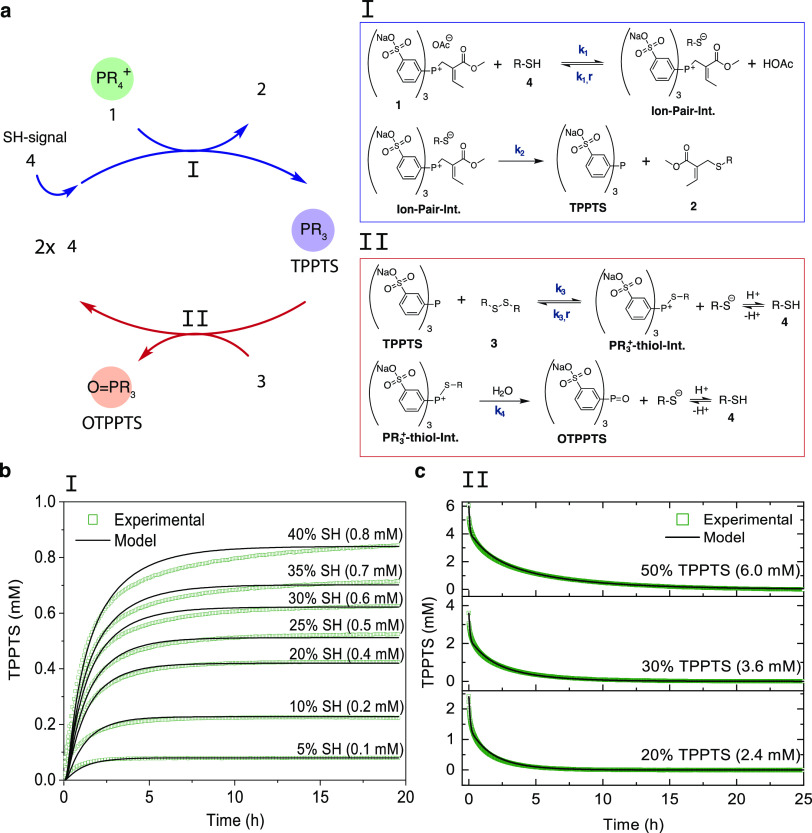
Nucleophilic substitution and disulfide reduction kinetics. (a) Schematic representation and full reaction pathway overview for nucleophilic substitution (I) and disulfide reduction (II) in the amplification system. (b) SH-triggered substitution kinetics of compound **1** (2.0 mM), measured by the appearance of TPPTS from UV–vis at 260 nm. (c) Disulfide reduction kinetics for **3** (12.0 mM) at varying concentrations of TPPTS, measured by the disappearance of TPPTS from UV–vis at 300 nm. All reactions were measured in 0.1 M phosphate buffer (pH = 7.6) at 25 °C. Representative samples from duplicate runs (green line). Solid black lines correspond to the model fits to each condition. Statistical evaluation between the kinetic model and experimental data can be found in Supporting Figures 13–16.

Prior to the experimental investigation of the entire amplification system, we conducted kinetic experiments for both (I) thiol-triggered substitution of **1** using **4** and (II) disulfide reduction of **3** by TPPTS (Figure 3a). A simplified mathematical model was developed based on a set of nonlinear differentials describing (I) and (II) and solved numerically for a series of reactions. To begin, we used the experimentally determined pseudo-first-order rate constants for each model, which were obtained using UV–vis experiments (Supporting Figure 12a,b). By implementing the existence of intermediate species based on the mechanism of the nucleophilic substitution through phosphonium ion intermediates proposed by Krische and co-workers^40^ (Figure 3a-I), we found good agreement (*R*^2^ = 0.89–0.97) between the model and our experimental data (Figure 3b). We noticed, however, that the model overestimated the TPPTS release at a later stage in the reaction, which becomes more pronounced with increasing SH concentrations.

We described the disulfide reduction kinetics using a two-step mechanism.^41,42^ In the first step, the nucleophilic attack by phosphine forms one equivalent of thiolate anion (S^–^) and the S-alkylphosphonium ion adduct (PR_3_^+^-thiol-intermediate). The intermediate ion then undergoes subsequent hydrolysis to afford phosphine oxide and a second equivalent of S^–^ (Figure 3a-II). Applying this mechanism to our conditions, we were able to optimize reaction constants for all TPPTS variations, which resulted in excellent agreement (*R*^2^ = 0.99) between model prediction and experimentally acquired UV–vis data (Figure 3c). However, further mechanistic insight is required to enhance the predictive capabilities and scope of applicability of this simplified model. Most importantly, describing each reaction step accurately in separate submodels turns out to be a powerful means for individual reaction step prediction in signal amplification systems. However, the standard approach to develop these submodels by using progression fitting is only suboptimal since the combined system and the interactions between species are not accounted for. For example, the underlying short-lived species interactions, such as ion-pair interactions, clusters, and/or electrostatic interactions, are not well understood from separate reaction modeling and are often cumbersome to determine experimentally. This challenge can be partially overcome by turning toward model optimization. Optimization or re-evaluation of early on established reaction constants will be fundamental for signal amplification modeling to establish first understanding and at the same time to identify potential species interactions, which are unexpected or have never been observed beforehand.

### Signal Amplification: Small Molecule Study

**Figure 4:**
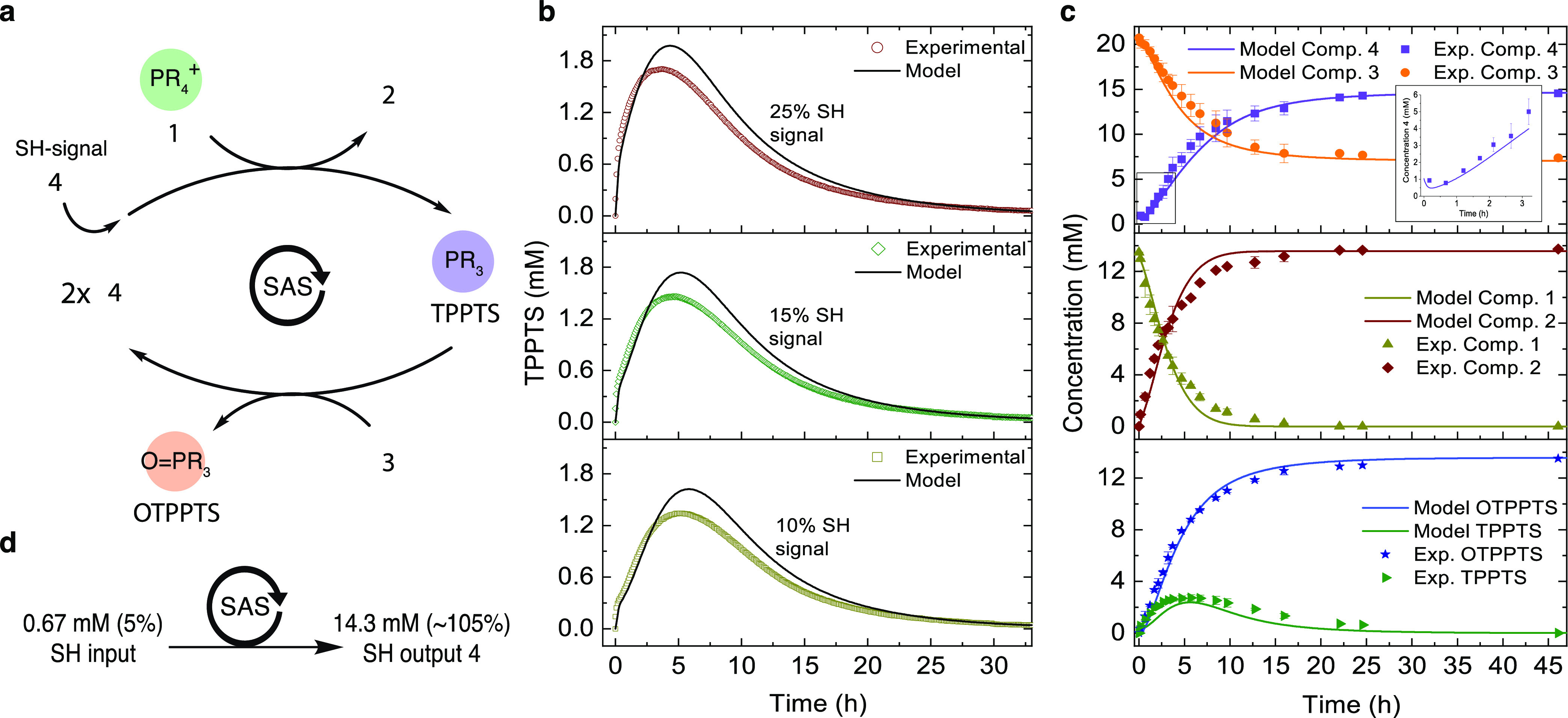
Kinetic experiments of the signal amplification system and model predictions for species variation. (a) Schematic representation and full reaction pathway overview for nucleophilic substitution and disulfide reduction in the amplification system. (b) UV–vis kinetic experiments using compound **4** as SH-trigger, at concentrations of 0.95 mM (10%), 1.40 mM (15%), and 2.30 mM (25%). Conditions: 9.0 mM of compound **1** with 13.5 mM of **3** in phosphate buffer (pH = 7.6) at 25 °C. (c) NMR kinetic experiments using 13.5 mM of **1** (1.0 equiv), 1.5 equiv of disulfide **3**, and 0.05 equiv of **4** in aqueous buffer (2:8 D_2_O: phosphate buffer (0.1 M, pH = 7.6)) at 25 °C. Error bars represent standard deviation from duplicate runs. Solid lines correspond to the model fits to each varying condition or species. (d) Schematic representation of the signal amplification system output using 0.05 equiv of **4**.

Initially, we studied the kinetics of the signal amplification system by tracking the UV–vis absorbance changes of TPPTS. The amplification experiment was performed at optimized conditions with 9.0 mM of phosphonium ion **1** (1.0 equiv) and 1.5 equiv of disulfide **3**. We used thiol signal **4** as a model trigger for the system (Figure 4a). When adding SH-trigger to the mixture**,** the release of TPPTS was monitored at λ = 300 nm upon conversion of **1** and the subsequent oxidation of released TPPTS by **3**. We monitored the kinetics of the reaction cascade for different ratios of SH-trigger (0.10, 0.15, and 0.25 equiv) (Figure 4b). Additionally, we used the kinetic model to describe the reaction and quantified the fit between the model and experimental data by determining the coefficients of determination *R*^2^.^43^

From the kinetic experiments, we found that the TPPTS concentration increased with increasing SH-signal. As expected, the release of TPPTS (*k*_4_ = 0.0020 M^–1^ s^–1^) is approximately three times as fast as the oxidation of TPPTS in the presence of **3** (*k*_4_ = 0.00071 M^–1^ s^–1^) based on their rate-determining step rate constants. As seen from Figure 4b, once the trigger is applied, the initiation speed and the maximum concentration of TPPTS from 0 to 5 h differ notably (0–5 h), while TPPTS depletion (5–35 h) propagates at approximately the same rate. Using the kinetic model, we were able to predict the signal amplification cascade for concentrations of 10, 15, and 25% SH-triggers with *R*^2^—values of 0.91, 0.92, and 0.93, respectively. Although the model is in good agreement with experimental data, it overestimates the amplitudes of released TPPTS in all three cases. An explanation for the discrepancy could be the influence of electrostatic interactions between **1**, TPPTS, and OTPPTS, which lead to lower reactivities during consumption, which have not been considered in our model. Importantly, however, these results confirmed that the amplification cascade detects and translates the amount of trigger, causing signal-dependent amplitude curves of released TPPTS and time-dependent consumption of **1**.

Next, we investigated the reaction cascade using ^1^H NMR to examine all species, including the amplification of the SH-trigger analyte **4**. First, we explored the background reaction (no thiol **4**) using 13.5 mM of **1** (1.0 equiv) and 1.5 equiv of **3** in aqueous buffer. The experiment revealed no conversion of **1** without signal initiation after 24 h (Supporting Figure 5), which is a highly desirable feature for an amplification reaction.^3^ This result confirms our earlier findings using **1** in DMEM (cell culture media) containing cystine disulfide (0.2 mM).

Initiating the system using **4** (Figure 4c), we found that during the time course of the reaction cascade by ^1^H NMR (Supporting Figure 11), compound **4** exhibits a sigmoidal amplification profile, which is a typical attribute for autoamplification systems.^3^ In particular, the use of 0.67 mM SH-signal (∼5%) to initiate the reaction resulted in a thiol concentration increase to approximately 14.3 mM (∼105%) after 45 h, accounting for the full conversion of **1** (Figure 4c-top,d). Matching this process, the disulfide concentration quantitatively decreases ∼13.5 mM from 20.3 to 7.4 mM. Simulations using the kinetic model for **3** (*R*^2^ = 0.93) and **4** were consistent with the experimental data. Importantly, the model was able to describe the sigmoidal amplification profile of **4** in detail (Figure 4c, inset) and with high accuracy (*R*^2^ = 0.96). Furthermore, we found that the production of **2** corresponds to the conversion of **1** (Figure 4c-middle). Here, our simulations substantiate the exponential decrease of **1** and an increase of **2**, with *R*^2^-values of 0.98 (**1**) and 0.96 (**2**). Similarly, the OTPPTS concentration is in excellent agreement with simulation results (*R*^2^ = 0.98) (Figure 4c-bottom). Using ^1^H NMR spectroscopy to track all species involved in the two-component reaction cycle and describing those with kinetic simulations, we were able to confirm the successful amplification of thiol **4** through the coupled nucleophilic substitution and disulfide reduction reactions.

### Naked-Eye SH-Analyte Detection through Signal-Amplified Hydrogel Degradation

**Figure 5:**
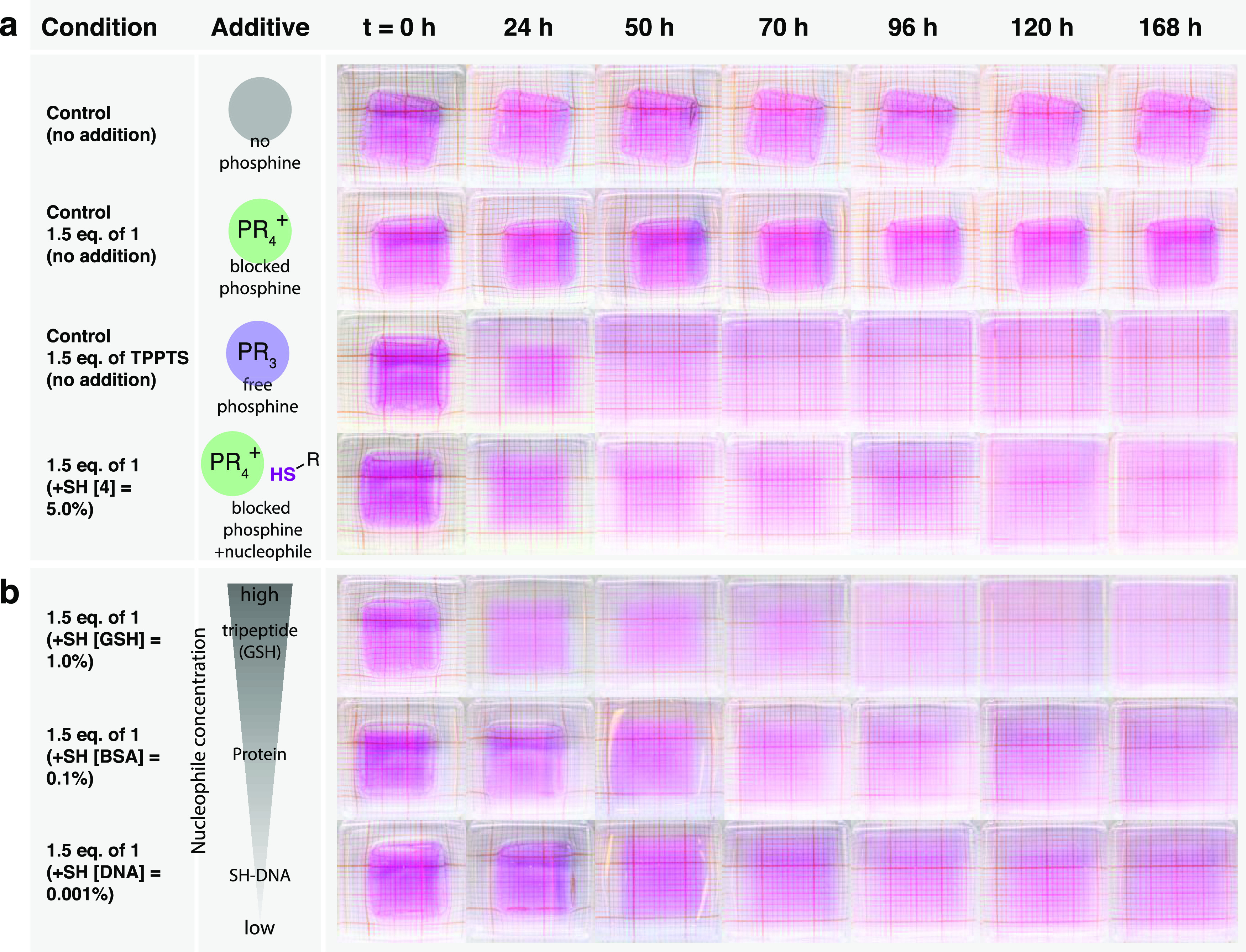
Time lapse photographs of hydrogel degradation using the amplification system triggered by SH-analytes. (a) Control gels with (1) no additives, (2) with 1.5 equiv of **1**, (3) 1.5 equiv of TPPTS, and (4) 1.5 equiv of **1** and 5% (0.05 equiv) of **4**. (b) Gels with 1.5 equiv of **1** and SH-trigger addition of (1) 1.0% (0.01 equiv) of l-glutathione, (2) 0.01% (0.0001 equiv) of bovine serum albumin, and (3) 0.001% (0.00001 equiv) of thiol-functionalized DNA. Conditions: gels were submerged in 1.5 mL of phosphate buffer (0.1 M, pH = 7.6). All measurements were done in duplicate (see Supporting Figures 17 and 18).

Once we established the signal amplification strategy and obtained their model kinetics, we then sought to incorporate this strategy into a macromolecular-cross-linked hydrogel system for naked-eye SH-analyte detection. We cross-linked DMA with BAC to form cube-shaped polymer hydrogels with a cross-linker concentration of 4.6 mg/mL (3.5 wt % cross-linker). The obtained gels had a water content of 98 ± 0.81 wt %, dimensions of approximately 1.3 cm × 1.2 cm × 0.5 cm (L/W/H), and had been copolymerized with methacryloxyethyl thiocarbamoyl rhodamine B (0.001 wt %) as a color indicator to enable the visualization of hydrogel degradation (Figure 5). To study the self-amplified material degradation process via dissolution of the polymer gels, we first conducted experiments by exposing hydrogels submerged in aqueous buffer (phosphate buffer, 0.1 M, pH = 7.6) with 1.5 equiv of **1** (vs cross-linker) to 0.05 equiv (5%) of **4** (vs compound **1**) as a model SH-trigger. We observed gradual gel dissolution over the course of 168 h, while no degradation occurred without thiol initiation (controls: only compound **1** without SH-trigger and no additives) (Figure 5a). Note that although no degradation on gels occurred in the absence of trigger during the observation period of 168 h, we found that gels started to degrade beyond approx. 13 days being exposed to air and light (data not shown).

Hydrogel degradation is based on the concept that, after the addition of **4**, the self-propagating reaction with **1** successfully releases TPPTS within the hydrogel matrix, where it is free to diffuse and propagate the cascade through phosphine-mediated disulfide reduction on a cross-linker, generating new thiols (new signals). The progression of the self-propagation continues until the complete physical degradation of the material (*t* = ∼168 h). In comparison, hydrogels exposed to TPPTS alone (1.5 equiv vs cross-linker) showed visibly faster degradation than the thiol-triggered amplification system gels by complete dissolution within 50 h.

Next, we initiated the system using different thiol triggers. We focused on biologically relevant thiol analytes comprised of various sizes, including l-glutathione (SH-GSH), the protein bovine serum albumin (SH-BSA), and thiol-functionalized DNA (SH-DNA) with concentrations of 132, 1.32, and 0.132 μM, respectively. Importantly, each SH-trigger (SH-BSA,^44^ SH-GSH, and SH-DNA) contains one thiol equivalent per molecule. Note that SH-BSA has additional disulfide bridges, which account for an additional 27% of disulfides in the hydrogel system. We hypothesized that variations in the concentration of SH-trigger would lead to a differential self-propagation speed and signal amplification rate, which could be observed by the naked eye. Samples containing SH-DNA (0.001% SH vs **1**) showed slower decomposition than samples containing SH-BSA (0.01% SH) and SH-GSH (1.0% SH) (Figure 5b). The gel degradation rate increases with an increasing concentration of SH-trigger due to a higher TPPTS release at the start. As a result, the signal amplification rate of the system is increased according to SH-GSH > SH-BSA > SH-DNA (Figure 5b). Remarkably, the visual degradation between SH-BSA and SH-DNA is less than expected although the SH-BSA signal concentration is 10-fold larger compared to SH-DNA. We suspect that the 27% additional disulfides present in SH-BSA affect the overall degradation rate, which results in slower gel decomposition time.

Interestingly, we observed that SH-GSH (1.0%) showed a faster decomposition than SH-trigger **4** (5%). We rationalized this behavior to be related to the higher nucleophilicity^45^ of glutathione than that compared to **4**, which accelerates decomposition (Figure 5a,b) and overshadows over time their concentration difference.

### Damage-Triggered Hydrogel Destruction through Cut-Generated Radical Initiation

Opening of sulfur cross-links in soft materials (e.g., hydrogels) has been frequently realized in the presence of reducing agents (e.g., trivalent phosphorous reagents^46^) and through thiols via thiol–disulfide exchange,^47^ whereas examples in which mechanical stress or force^48^ is used as a trigger are rare. To further evaluate our system, we were interested to see if compound **1** is capable of sensing mechanical impact through damage-generated radicals and subsequent TPPTS release to initiate the amplification cascade. Applying mechanical stresses on polymers and hydrogel networks is well-known to cause polymer chain cleavage at the C–C backbone bonds and the formation of radicals.^49−51^ Similarly, mechanical-induced forces on disulfide cross-linked hydrogel networks likely result in homolytic disulfide scission, leading to thiyl radicals.^48,52,53^ These radicals then form thiols by abstracting hydrogens from water^53^ or other donor molecules.^54^ These newly formed thiols can then initiate the amplification system by releasing TPPTS from **1**.

**Figure 6:**
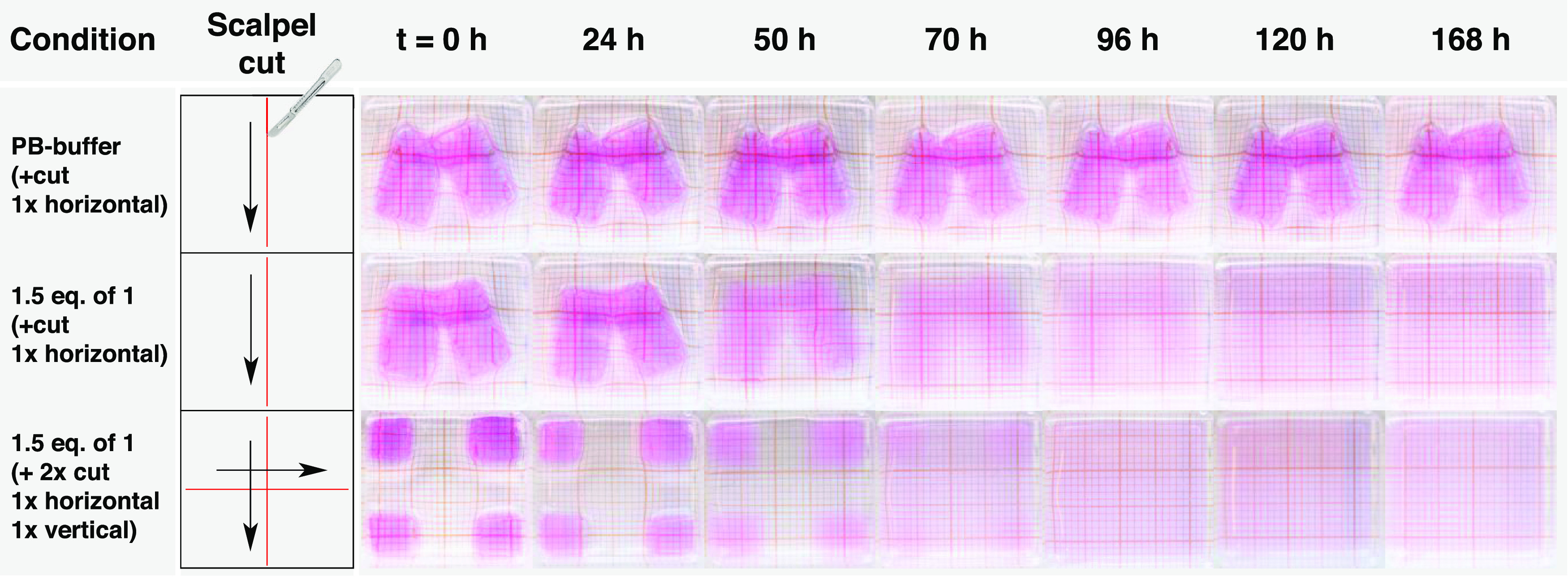
Time lapse photographs of hydrogel degradation using the self-amplification system triggered by damage (cut). Control gels with (1) no additives and 1× horizontal cut, (2) 1.5 equiv of **1** and 1× horizontal cut, and (3) 1.5 equiv of **1** and 2× cut (1× horizontal and 1× vertical). Conditions: gels were submerged in 1.5 mL of phosphate buffer (0.1 M, pH = 7.6). All measurements were done in duplicate (see Supporting Figure 19).

To test this hypothesis and evaluate thiyl radicals as potential triggers, we conducted experiments on gels (1.5 equiv of **1** vs cross-linker) by applying force via cutting horizontally through the material using a scalpel and compared it to identical gels without **1** (Figure 6a). Consistent with earlier findings, we found that the applied damage can indeed induce the amplification cascade leading to complete degradation of the gels after 168 h. In contrast, gels without **1** remained stable during the entire observation time. To further confirm the effect of mechanical damage and to provide a correlation between material damage and its degradability, we carried out experiments for which we applied two cuts on the gels (1× horizontally and 1× vertically). Since more cuts generate more thiols, we hypothesized faster degradation due to higher activation of **1**. Indeed, from the comparison in Figure 6, it can be seen that gels that were cut two times degraded faster than gels with only one cut. In particular, single-cut gels show complete dissolution after 120 h, while two times cut gels became a homogeneous solution after 96 h. Importantly, thiol availability is likely not the sole contributor to hydrogel degradation. A two-cut zone creates a larger interface for TPPTS diffusion than compared to a one-cut zone, which ultimately enhances the accessibility of phosphines to disulfide cross-linkers. In general, these results indicate that the material is capable of sensing force-induced damage by translating the stimulus through the amplification system to respond to material degradation.

## Conclusions

We have developed a new signal amplification system for detecting thiol compounds using the reactivity of allylic phosphonium ion **1** with thiols and disulfides. Importantly, upon complete removal of thiols from the system, we observed no background interference from hydrolysis or unwanted site reactions over the course of 24 h. System activation by the allylic substitution of TPPTS from **1** only commences in the presence of thiol analytes. Here, we used substoichiometric amounts of thiol to initiate a chain reaction that exponentially amplifies the input thiol signal. Experimental data is supported by a kinetic model that accurately describes the rates of all species involved in the amplification cycle and predicts variations in individual components, providing further insight into the system.

Combining this amplification strategy with disulfide cross-linked hydrogel structures enabled us to detect multiple thiol analytes, including l-glutathione, a protein, and DNA, by visual hydrogel degradation. The system is highly sensitive to SH concentrations as low as 0.132 μM and across three orders of magnitude in concentration and can even react to force-generated signals. Despite these advances, the current system does have drawbacks, e.g., the reagents are not covalently linked to the material and the amplification process is slow. Nevertheless, this proof-of-concept for naked-eye detection is a promising step toward a new generation of responsive soft materials, such as coatings and adhesives, that can show an amplified response to low exposure of a specific applied stimulus, resulting in a macroscopic change in the material.

## Supporting Information Available

General information about materials, synthesis, and characterization procedures, control experiments (molecular stability, nucleophilic reactivity, and kinetics), computational model description, and hydrogel degradation study (PDF)
