# Supplementary material for: Naked-Eye Thiol Analyte Detection via Self-Propagating, Amplified Reaction Cycle
Source: J Am Chem Soc. 2023 Sep 25;145(39):21222–30. doi: 10.1021/jacs.3c02937 (PMC10557148; doi:10.1021/jacs.3c02937)
Supplement: Supplementary file 1 — ja3c02937_si_001.pdf [file ja3c02937_si_001.pdf]

Supplementary Information

Naked-eye thiol analyte detection via self-propagating, amplified reaction cycle

Benjamin Klemm[a], Ardeshir Roshanasan [a], Irene Piergentili[a], Jan H. van Esch and Rienk Eelkema[a,*]

[a] Delft University of Technology, Department of Chemical Engineering, Van der Maasweg 9, 2629 HZ Delft, The Netherlands.

* Correspondence to: R.Eelkema@tudelft.nl

Table of Contents

1.0 Materials and Methods 3
1.1 Instrumentation, materials and characterization 3
1.2 NMR spectroscopy 3
1.2.1 Fitting pseudo-first order reaction rate 3
1.3 Hydrogel preparation 4
1.3.1 Remaining thiol removal: post treatment of hydrogels 4
1.3.2 Water content of hydrogels 4
1.4 UV-vis spectroscopy 4
1.4.1 UV-vis experiments for nucleophilic substitution reaction 4
1.4.2 UV-vis experiments for disulfide reduction reaction 5
1.4.3 UV-vis experiments for amplification cycle reaction 5
1.4.4 Calibration lines 5
2.0 Molecular stability tests 6
2.1 NMR observation of compound 1 in buffer media 6
2.2 NMR observation of compound 1 in cell-culture media 7
2.3 NMR observation of TPPTS in buffer media 8
2.4 NMR observation of 1 and 3 in buffer media 9
3.0 Kinetic experiments of nucleophilic reactivity 10
3.1 NMR observation of reactivity of compound 1 with 20% L-glutathione signal (SH-nucleophile) 10
3.2 NMR observation of reactivity of compound 1 with 20% N-acetyl cysteine signal (SH-nucleophile) 11
3.3 NMR observation of reactivity of compound 1 with 20% L-proline signal (NH-nucleophile) 12
3.4 NMR observation of reactivity of compound 1 with 20% L-phenylalanine signal (NH_2-nucleophile) 13
3.5 NMR observation of reactivity of compound 1 with 20% p-nitrophenol signal (O-nucleophile) 14
4.0 Kinetic experiments of signal-amplification cycle 15
4.1 NMR observation of signal-amplification cycle with 5% signal 15
5.0 Kinetic model 16
5.1 UV-vis spectroscopy – rate constant determination 16
5.2 Forward reaction: TPPTS release modelling 18
5.3 Backward reaction: disulfide reduction modelling 21
5.4 Signal-amplification cycle - model 24
6.0 Hydrogels – signal-amplification experiments 25
6.1 Photo-observation of signal-amplified hydrogel degradation study 25
7.0 Synthesis of compounds 27
7.1 Synthesis of trisodium 3,3',3''-((2-(methoxycarbonyl)but-2-en-1-yl)phosphoniotriyl)tribenzenesulfonate) acetate (1) 27
7.2 Synthesis of methyl 2-(((2-acetamidoethyl)thio)methyl)but-2-enoate (2) 27
7.3 Synthesis of N,N-diacetylcystamine (3) 27
7.4 Synthesis of N,N- Bis (acryloyl) cystamine (BAC) 28
7.5 Synthesis of tris(sodium-m-sulfonatophenyl)phosphanoxide (OTPPTS) 28
8.0 NMR Spectra 29
9.0 2-D NMR Spectra 33
10.0 LC-MS data 36
11.0 References 37

1.0 Materials and Methods

1.1 Instrumentation, materials and characterization

All reagents and solvents were used without further purification unless otherwise stated. Methyl 2-(1-acetoxyethyl)acrylate (acrylate-1, 98%), triphenylphosphine-3,3′,3″-trisulfonic acid trisodium salt (TPPTS, ≥95.0%), N-acetylcysteamine (**4**, 95%), N,N-dimethylacrylamide (DMA, 99%), cystamine dihydrochloride (≥98.0%), acetic anhydride (≥99%), methacryloxyethyl thiocarbamoyl rhodamine B, tetramethylethylenediamine (TEMED, 99%), ammonium persulfate (APS, 98%) and DMEM (Dulbecco's Modified Eagle Medium, Thermo Fisher 11971-025) were purchased from Sigma Aldrich, TCI Europe, Thermo Fisher Scientific or Polysciences Inc. For the preparation of aqueous buffers, solid salts were used: sodium phosphate monobasic and sodium phosphate dibasic, purchased from Sigma Aldrich. Unless stated otherwise, all stock solutions were prepared in D_2O/phosphate buffer mixture 2:8 (0.1 M, pH = 7.6). All buffers were pH adjusted using sodium hydroxide (1 M) and hydrochloric acid (1 M). DMA was passed through basic alumina prior to use to remove inhibitor. ESI-MS was performed using LTQ XL spectrometer equipped with Shimadzu HPLC setup operating at 0.2 mL/min flow rate with water/MeCN mobile phase containing 0.1 vol% formic acid and Discovery C18 column. Photographs were taken on a Canon EOS 600D single reflex camera with a Canon Macro Lens EF 100 mm 1:2.8 USM. UV-vis spectroscopic experiments were performed with an Analytik Jena Specord 250 spectrophotometer using quartz cuvettes with a 1 mm path length, a volume of 0.4 mL and a temperature controller set to 25°C.

1.2 NMR spectroscopy

NMR spectra were recorded on an Agilent-400 MR DD2 NMR instrument at 25°C (399.7 MHz for ^{1}H, 100.5 MHz for ^{13}C and 161.9 MHz for ^{31}P) using residual solvent signals as internal reference. Sodium trimethylsilylpropanesulfonate (DSS) was used as internal standard for NMR kinetic experiments with reference resonance at 0.0 ppm. To suppress the water peak, PRESAT or ES_suppression configuration (suppress one highest peak) was used. NMR spectra were processed by MNova NMR software (Mestrelab Research).

1.2.1 Fitting pseudo-first order reaction rate

The pseudo-first order reaction rate constants were determined by fitting the conversion of **1** ([B]t) over time with the following equation:

$$\ln\left(\frac{[B]_t}{[B]_0}\right) = -k[A]_0 * t \quad \text{Eq.1}$$

$[B]_0$ = initial concentration of **1** at t = 0, 0.067 mM (1.0 eq.); $[B]_t$ = the concentration of **1** at every time point obtained from ^{1}H NMR, with DSS as the standard; *k* is the pseudo-first order reaction rate constant ($M^{-1}*h^{-1}$) and $[A]_0$ = initial concentration of nucleophile (0.2 eq.).

1.3 Hydrogel preparation

DMA–BAC hydrogels (3.5% wt%) were synthesised by free radical polymerization. In all cases monomer mole ratios were BAC: DMA:Methacryloxyethyl thiocarbamoyl rhodamine B of 1:14:0.001. Typically, DMA (110 mg, 1.1 mmol) and BAC (20 mg, 0.08 mmol) were dissolved in deionized water (2.9 mL). Stock solutions of methacryloxyethyl thiocarbamoyl rhodamine B (50 µL, 0.04 mg, 1 µmol in DMSO) and TEMED (50 µL, 14.2 mg, 0.12 mmol) were prepared and added to the reaction mixture. Nitrogen was bubbled through the solution for 2 minutes. The polymerization was initiated by addition of APS solution (50 µL, 14 mg, 0.06 mmol) and quickly added to a mould where the mixture was allowed to proceed at room temperature. Gel formation occurred within 1 h. The hydrogels were removed from their mould and dialyzed for 48 hours against water.

1.3.1 Remaining thiol removal: post treatment of hydrogels

To eliminate any remaining thiols within the gel matrix, the gels were submerged in 1 mL of phosphate buffer (0.1 M, pH = 7.6) containing acrylate-1 (5.0 mg/mL). The gels were left in solution for 8 hours to react and hereafter were dialyzed for 48 hours against water before further usage.

1.3.2 Water content of hydrogels

The water content of the hydrogels was determined by using the gravimetric method. The wet weight (W_w) was measured after removing surface moisture of the hydrogel by wiping with a lens cleaning paper. The hydrogels were then dried in a drying oven for 24 hours at 50°C. Hereafter, the dried gels were weighed and the dry weight (W_d) was recorded. The water content was calculated according to Eq. 2:

$$water\ content\ (\%) = \frac{W_w - W_d}{W_w} * 100 \qquad \text{Eq.2}$$

Measurements were performed in duplicate and the results of water content was expressed as the mean ± standard deviation.

1.4 UV-vis spectroscopy

1.4.1 UV-vis experiments for nucleophilic substitution reaction

Stock solutions of compound **1** and **4** were prepared in phosphate buffer (0.1 M, pH = 7.6). The experiments were performed using 2.0 mM of **1** (0.74 mg, 1.0 eq.) and varying concentration of **4** (0.05, 0.10, 0.20, 0.25, 0.30, 0.35 and 0.40 eq.). The stock solution of **1** was added first to the UV-cuvette, followed by the addition of **4**, then shaken for 5 seconds and hereafter placed immediately in the UV-vis spectrophotometer for analysis. UV-vis spectra

were recorded at wavelength of 260 nm every 30s for 19 hours at a constant temperature (set to 25°C).

1.4.2 UV-vis experiments for disulfide reduction reaction

Stock solutions of compound **3** and TPPTS were prepared in phosphate buffer (0.1 M, pH = 7.6). The experiments were performed using 12.0 mM of **3** (1.42 mg, 1.0 eq.) and varying concentration of TPPTS (0.2, 0.3 and 0.5 eq.). The stock solution of **3** was added first to the UV-cuvette, followed by the addition of TPPTS, then shaken for 5 seconds and hereafter placed immediately in the UV-vis spectrophotometer for analysis. UV-vis spectra were recorded at wavelength of 300 nm every 30s for 25 hours at a constant temperature (set to 25°C).

1.4.3 UV-vis experiments for amplification cycle reaction

Stock solutions of compound **1**, **3** and **4** were prepared in phosphate buffer (0.1 M, pH = 7.6). The experiments were performed using 9.0 mM of **1** (3.33 mg, 1.0 eq.), 13.5 mM of **3** (1.6 mg, 1.5 eq.) and varying concentration of **4** (0.10, 0.15 and 0.25 eq.). The stock solution of **1** and **3** was added first to the UV-cuvette, followed by the addition of **4**, then shaken for 5 seconds and hereafter placed immediately in the UV-vis spectrophotometer for analysis. UV-vis spectra were recorded at wavelength of 300 nm every 30s for 36 hours at a constant temperature (set to 25°C).

1.4.4 Calibration lines

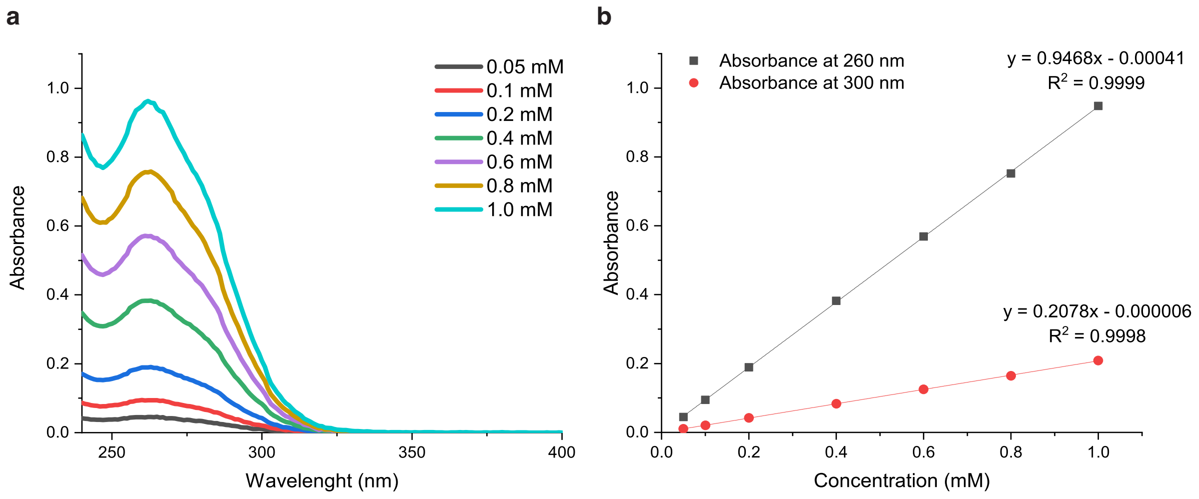

Supplementary Figure 1: Extinction coefficient for TPPTS in phosphate buffer (0.1 M, pH = 7.6) at 260 nm: 4.74 $mM^{-1}cm^{-1}$ and at 300 nm: 1.04 $mM^{-1}cm^{-1}$. (a) UV-vis absorbance spectra of TPPTS at different concentrations. (b) Absorbance at 260 and 300 nm of TPPTS at different concentrations.

2.0 Molecular stability tests

2.1 NMR observation of compound 1 in buffer media

Compound **1** (5.0 mg, 6.75 µM, 1.0 eq.) and DSS internal standard (1.47 mg, 1.0 eq.) were dissolved in 0.5 mL D_2O/phosphate buffer (2:8, 0.1 M, pH = 7.6). The reaction was followed by ^1H NMR spectroscopy for 24 hours.

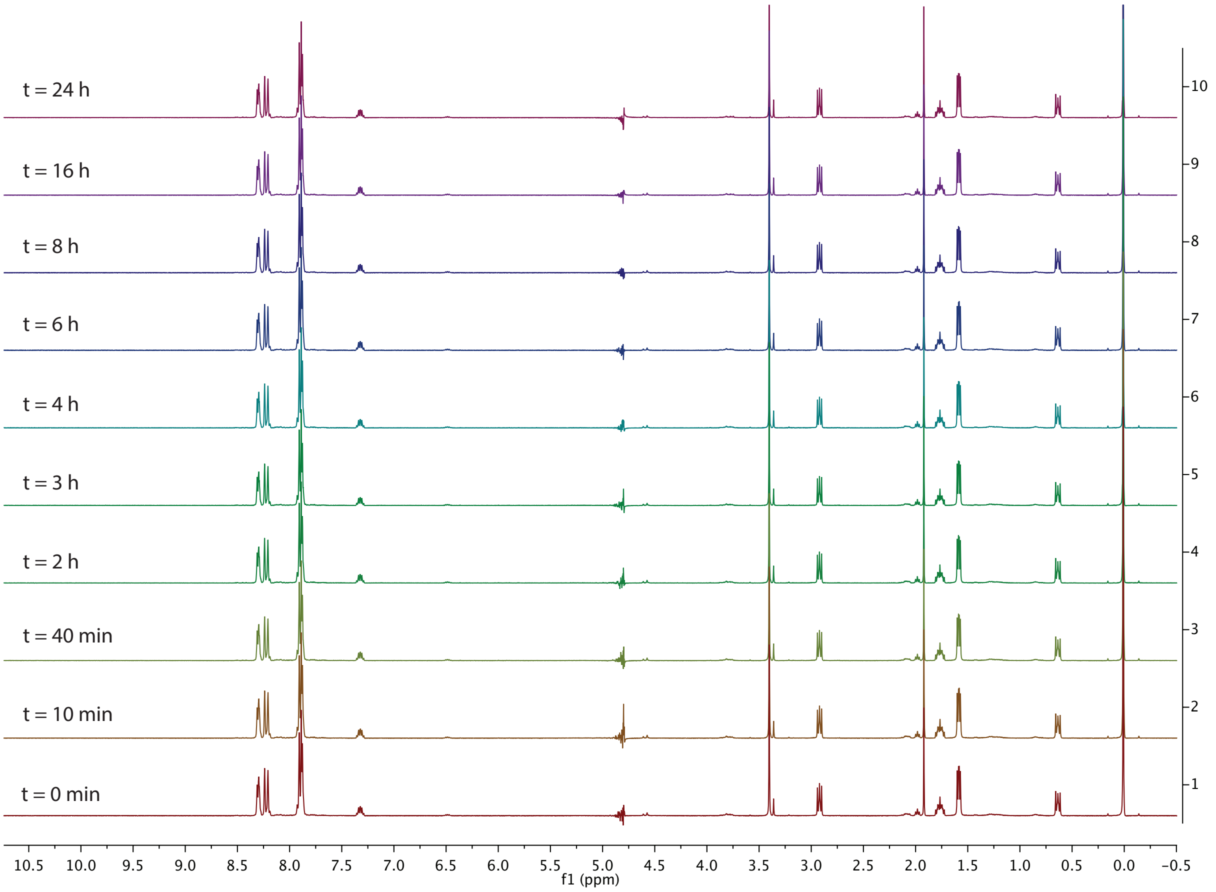

Supplementary Figure 2: Stability observation of **1** in ^1H NMR at different time points for 24 hours. The reaction was carried out in D_2O/phosphate buffer mixture 2:8 (0.1 M, pH = 7.6). The peak attributed to ~ 0.0 ppm corresponds to DSS internal standard and was used to align the spectra.

2.2 NMR observation of compound 1 in cell-culture media

Compound **1** (5.0 mg, 6.75 µM, 1.0 eq.) and DSS internal standard (1.47 mg, 1.0 eq.) were dissolved in 0.5 mL D_2O/Dulbecco's Modified Eagle Medium (DMEM, high glucose, no phosphates) 2:8. The reaction was followed by ^{1}H NMR spectroscopy for 22 hours.

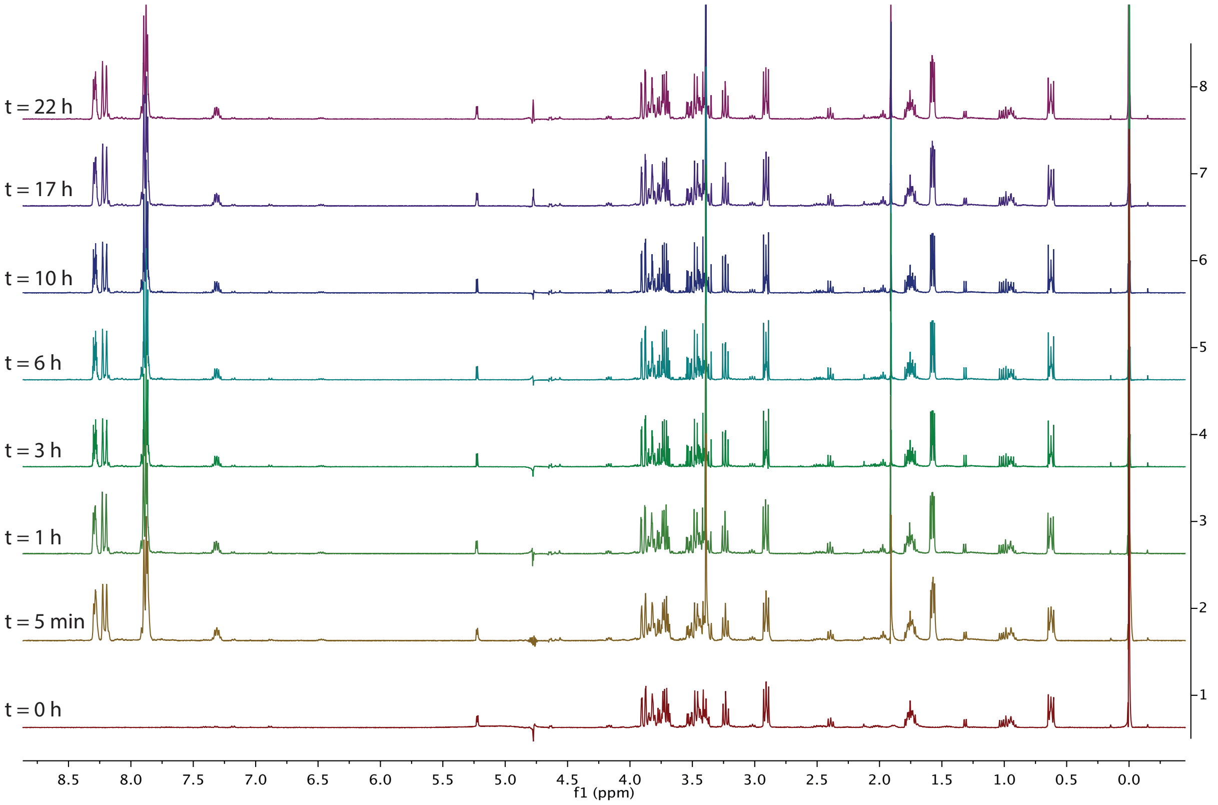

Supplementary Figure 3: Stability observation of **1** in ^{1}H NMR at different time points for 22 hours. The reaction was carried out in D_2O/Dulbecco's Modified Eagle Medium (2:8). The peak attributed to ~ 0.0 ppm corresponds to DSS internal standard and was used to align the spectra.

2.3 NMR observation of TPPTS in buffer media

TPPTS (3.84 mg, 6.75 µM, 1.0 eq.) and DSS internal standard (1.47 mg, 1.0 eq.) were dissolved in 0.5 mL D_2O/phosphate buffer (2:8, 0.1 M, pH = 7.6). The reaction was immediately followed by NMR for 24 hours.

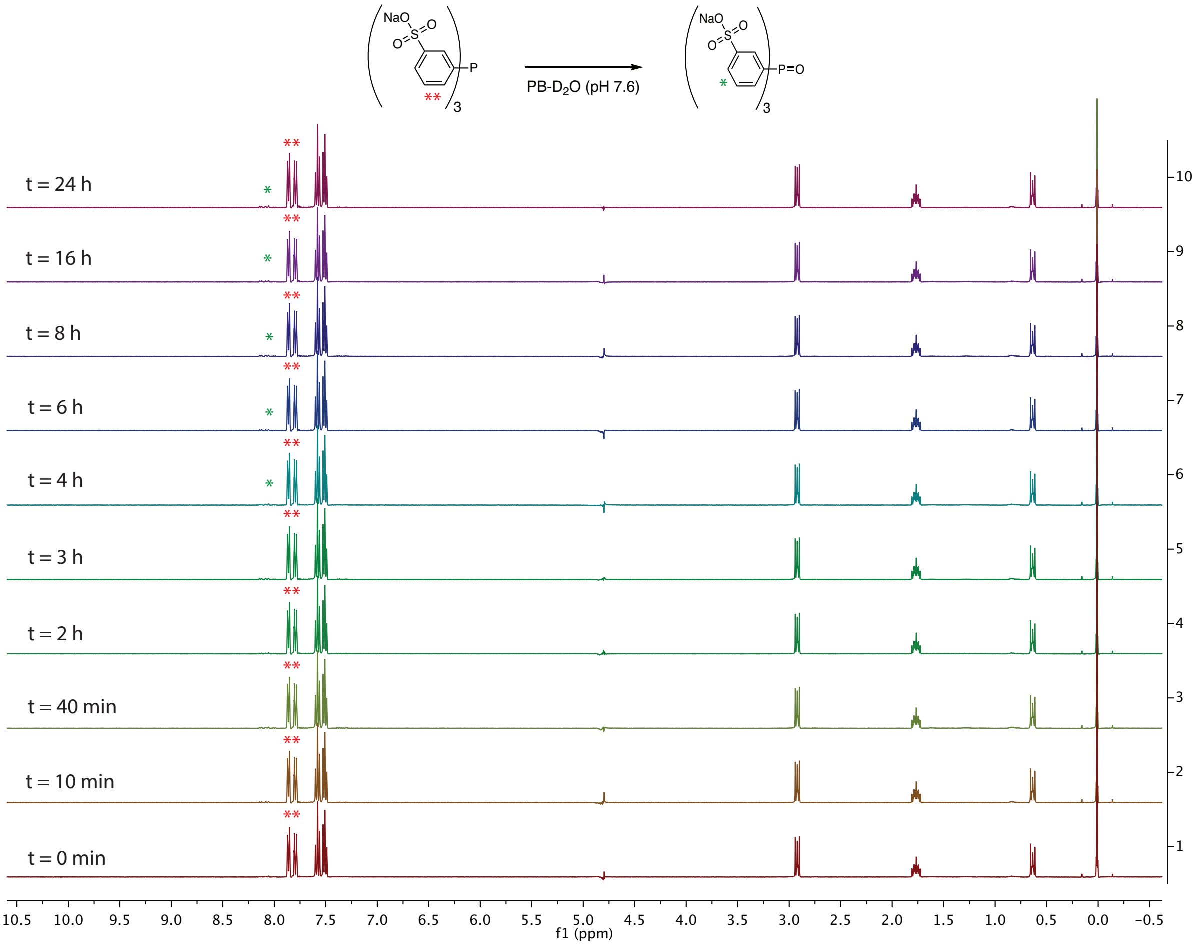

Supplementary Figure 4: Stability observation of TPPTS in ^{1}H NMR at different time points for 24 hours. The reaction was carried out in D_2O/phosphate buffer mixture 2:8 (0.1 M, pH = 7.6). The peak attributed to ~ 0.0 ppm corresponds to DSS internal standard and was used to align the spectra.

2.4 NMR observation of 1 and 3 in buffer media

Compound **3** (1.91 mg, 8.10 µM, 1.5 eq.), acrylate-1 (3.49 mg, 3.0 eq.) and DSS internal standard (1.47 mg, 1.0 eq.) were mixed in 0.4 mL phosphate buffer (pH = 7.6) and shaken for 30 minutes. To this solution compound **1** (5.0 mg, 1.0 eq.) in 0.1 mL H_2O was added and the mixture was shaken for another 30 minutes before freeze dried. The remains were solubilized in 0.5 mL (H_2O/D_2O 8:2). The final solvent conditions were 0.5 mL D_2O/phosphate buffer 2:8, 0.1 M, pH = 7.6). The reaction was immediately followed by ^1H NMR spectroscopy for 24 hours.

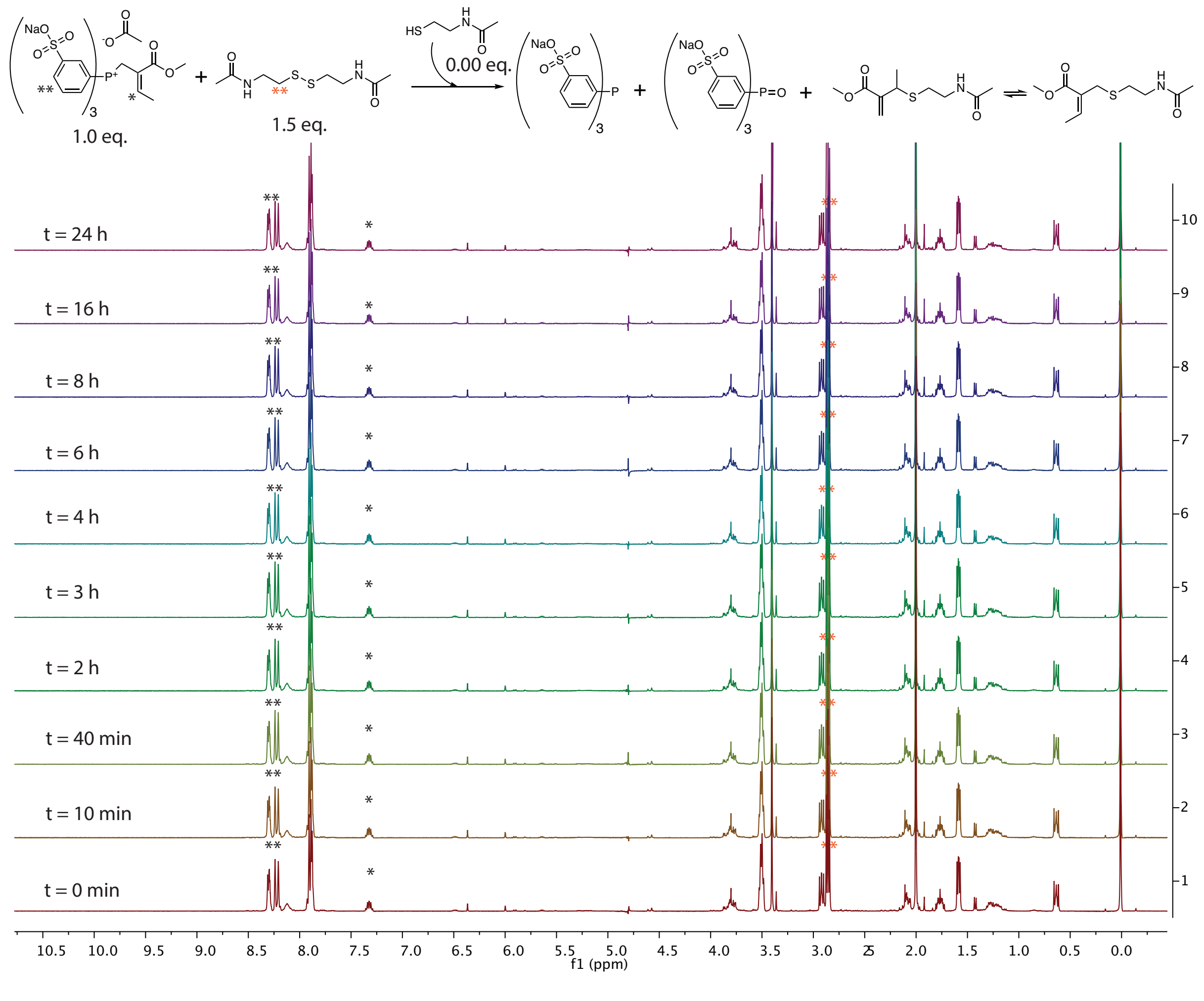

Supplementary Figure 5: Stability observation of compound **1** and **3** in ^1H NMR at different time points for 24 hours. The reaction was carried out in D_2O/phosphate buffer mixture 2:8 (0.1 M, pH = 7.6). The peak attributed to ~ 0.0 ppm corresponds to DSS internal standard and was used to align the spectra.

3.0 Kinetic experiments of nucleophilic reactivity

3.1 NMR observation of reactivity of compound 1 with 20% L-glutathione signal (SH-nucleophile)

Compound **1** (5.0 mg, 6.75 µM, 1.0 eq.) and DSS internal standard (1.47 mg, 1.0 eq.) were mixed in 0.4 mL D_2O/phosphate buffer 2:8, 0.1 M, pH = 7.6 and shaken. To this solution L-glutathione (0.41 mg, 0.2 eq.) in 0.1 mL phosphate buffer was added and the mixture was shortly shaken and immediately followed by ^1H NMR spectroscopy.

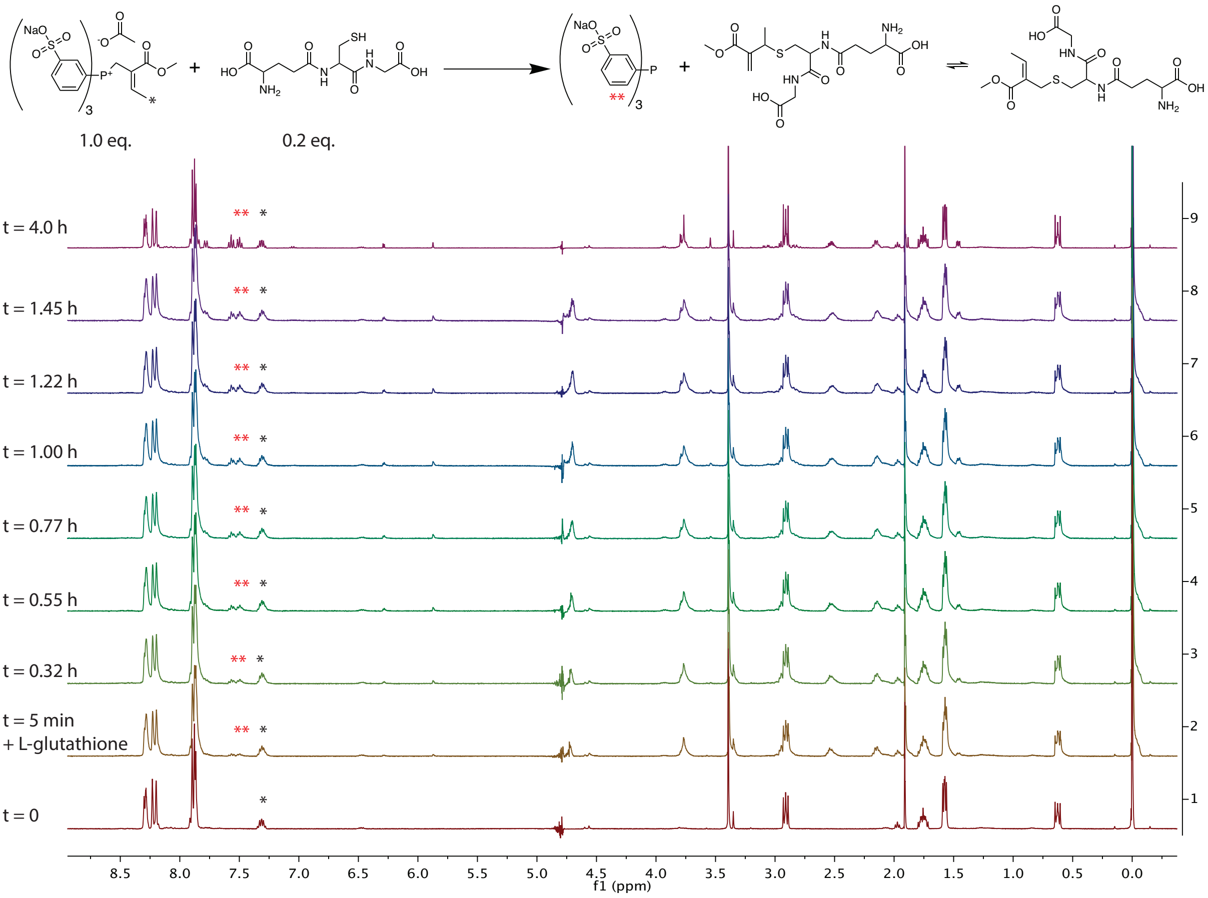

Supplementary Figure 6: Reactivity study of compound **1** with L-glutathione in ^1H NMR at different time points for 4 hours. The reaction was carried out in D_2O/phosphate buffer mixture 2:8 (0.1 M, pH = 7.6). The peak attributed to ~ 0.0 ppm corresponds to DSS internal standard and was used to align the spectra.

3.2 NMR observation of reactivity of compound 1 with 20% N-acetyl cysteine signal (SH-nucleophile)

Compound **1** (5.0 mg, 6.75 µM, 1.0 eq.) and DSS internal standard (1.47 mg, 1.0 eq.) were mixed in 0.4 mL D_2O/phosphate buffer 2:8, 0.1 M, pH = 7.6 and shaken. To this solution N-acetyl cysteine (0.23 mg, 0.2 eq.) in 0.1 mL phosphate buffer was added and the mixture was shortly shaken and immediately followed by ^{1}H NMR spectroscopy.

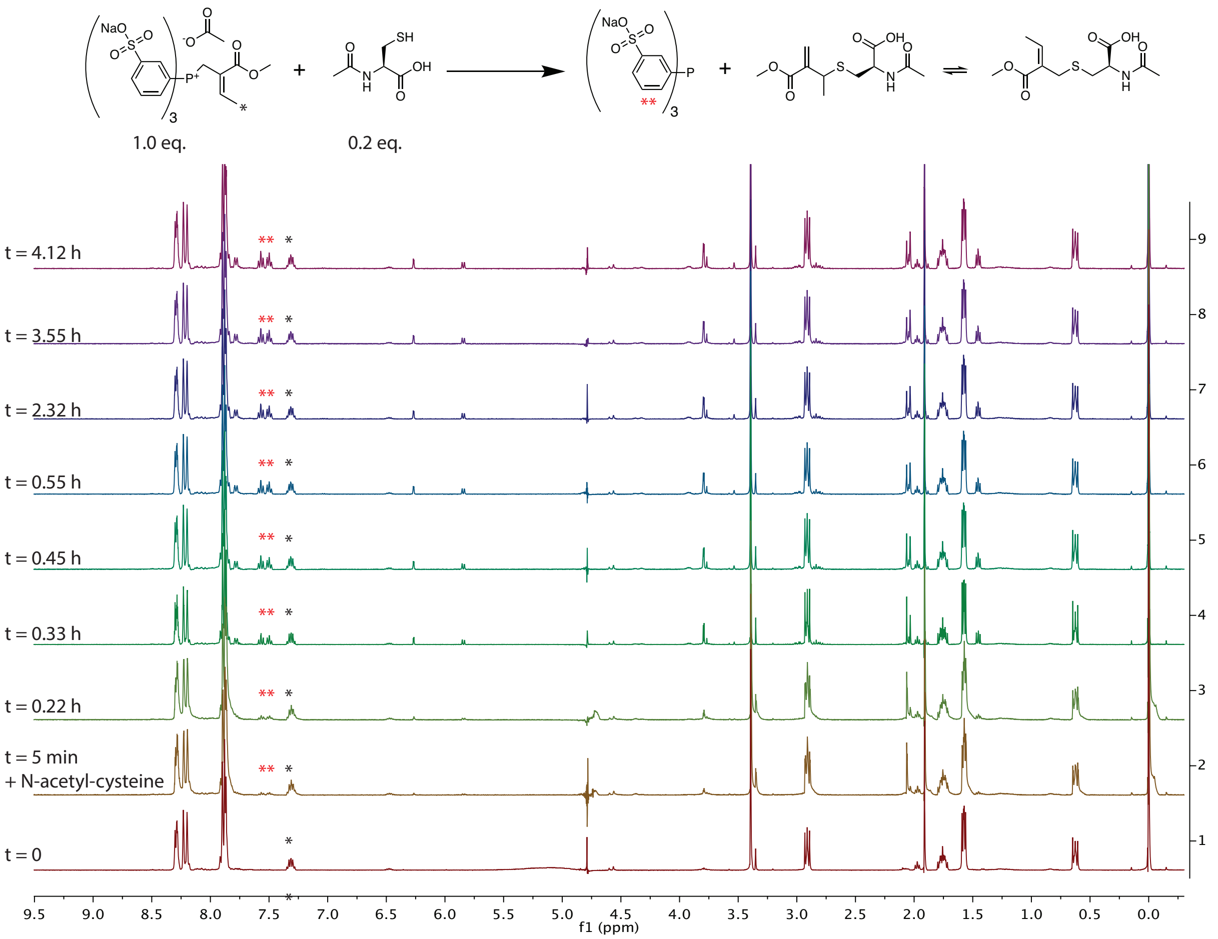

Supplementary Figure 7: Reactivity study of compound **1** with N-acetyl cysteine in ^{1}H NMR at different time points for 4 hours. The reaction was carried out in D_2O/phosphate buffer mixture 2:8 (0.1 M, pH = 7.6). The peak attributed to ~ 0.0 ppm corresponds to DSS internal standard and was used to align the spectra.

3.3 NMR observation of reactivity of compound 1 with 20% L-proline signal (NH-nucleophile)

Compound **1** (5.0 mg, 6.75 µM, 1.0 eq.) and DSS internal standard (1.47 mg, 1.0 eq.) were mixed in 0.4 mL D_2O/phosphate buffer 2:8, 0.1 M, pH = 7.6 and shaken. To this solution L-proline (0.13 mg, 0.2 eq.) in 0.1 mL phosphate buffer was added and the mixture was shortly shaken and immediately followed by ^{1}H NMR spectroscopy.

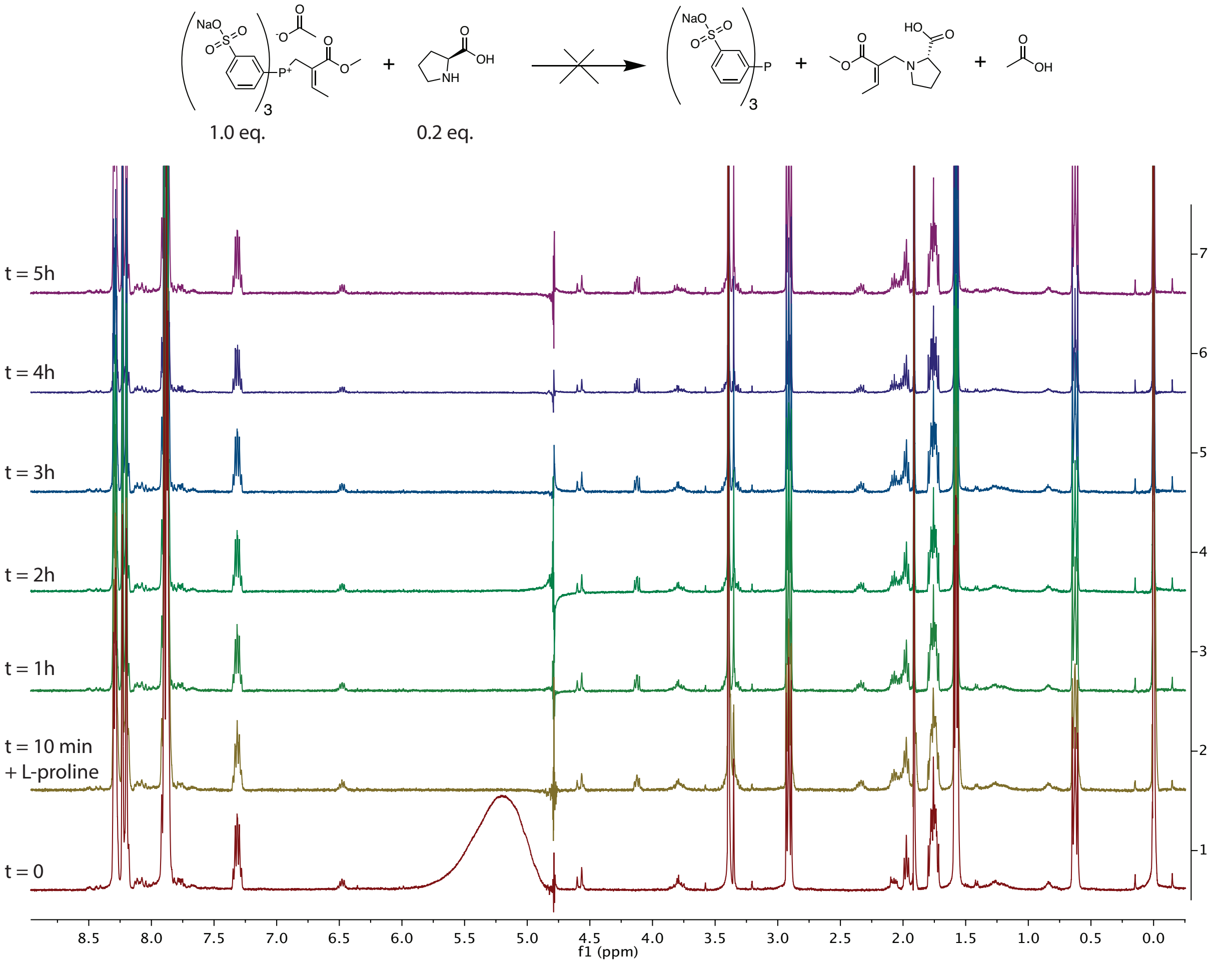

Supplementary Figure 8: Reactivity study of compound **1** with L-proline in ^{1}H NMR at different time points for 5 hours. The reaction was carried out in D_2O/phosphate buffer mixture 2:8 (0.1 M, pH = 7.6). The peak attributed to ~ 0.0 ppm corresponds to DSS internal standard and was used to align the spectra.

3.4 NMR observation of reactivity of compound 1 with 20% L-phenylalanine signal (NH_2-nucleophile)

Compound **1** (5.0 mg, 6.75 µM, 1.0 eq.) and DSS internal standard (1.47 mg, 1.0 eq.) were mixed in 0.4 mL D_2O/phosphate buffer 2:8, 0.1 M, pH = 7.6 and shaken. To this solution L-phenylalanine (0.16 mg, 0.2 eq.) in 0.1 mL phosphate buffer was added and the mixture was shortly shaken and immediately followed by ^{1}H NMR spectroscopy.

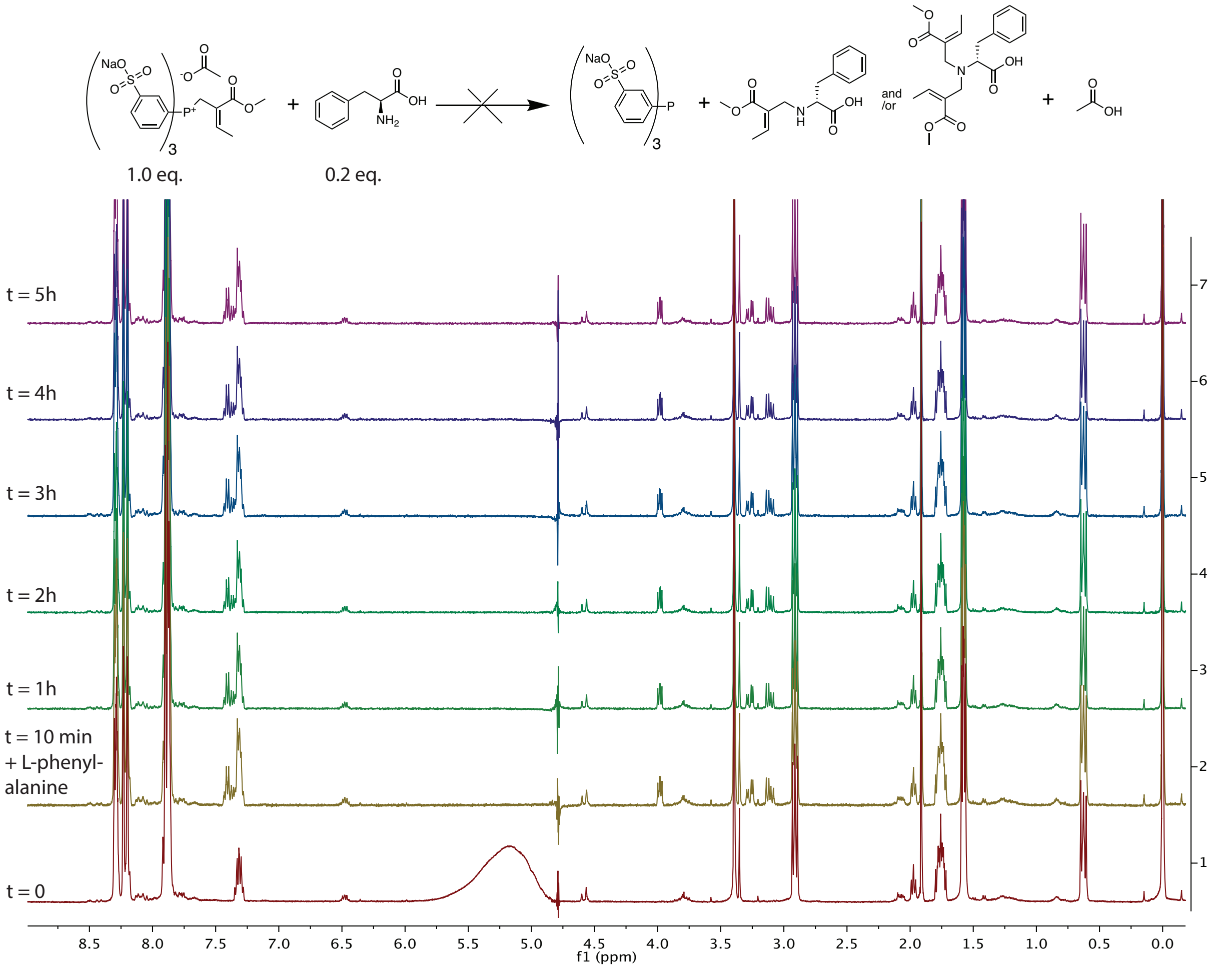

Supplementary Figure 9: Reactivity study of compound **1** with L-phenylalanine in ^{1}H NMR at different time points for 5 hours. The reaction was carried out in D_2O/phosphate buffer mixture 2:8 (0.1 M, pH = 7.6). The peak attributed to ~ 0.0 ppm corresponds to DSS internal standard and was used to align the spectra.

3.5 NMR observation of reactivity of compound 1 with 20% p-nitrophenol signal (O-nucleophile)

Compound **1** (5.0 mg, 6.75 µM, 1.0 eq.) and DSS internal standard (1.47 mg, 1.0 eq.) were mixed in 0.4 mL D_2O/phosphate buffer 2:8, 0.1 M, pH = 7.6 and shaken. To this solution p-nitrophenol (0.19 mg, 0.2 eq.) in 0.1 mL phosphate buffer was added and the mixture was shortly shaken and immediately followed by ^1H NMR spectroscopy.

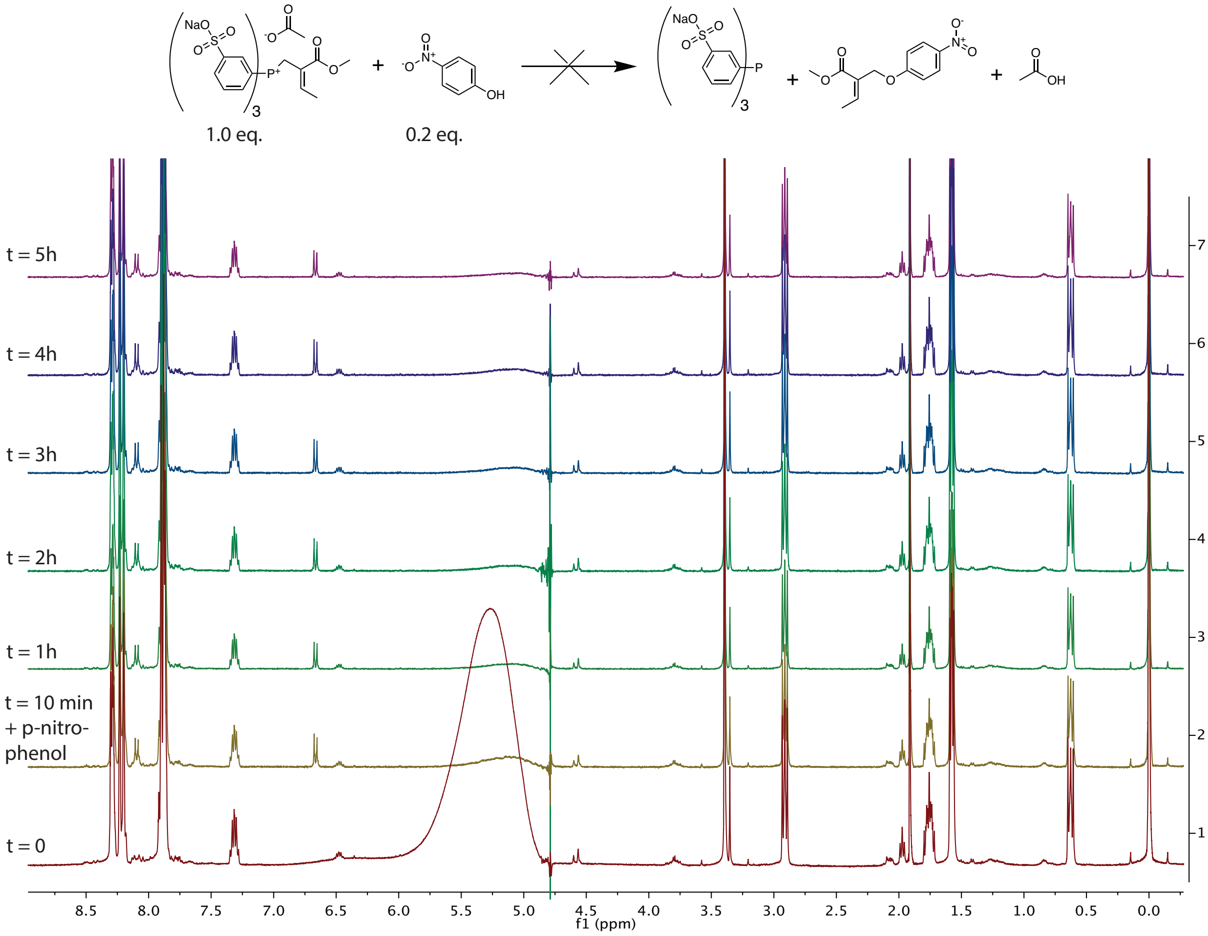

Supplementary Figure 10: Reactivity study of compound **1** with p-nitrophenol in ^1H NMR at different time points for 5 hours. The reaction was carried out in D_2O/phosphate buffer mixture 2:8 (0.1 M, pH = 7.6). The peak attributed to ~ 0.0 ppm corresponds to DSS internal standard and was used to align the spectra.

4.0 Kinetic experiments of signal-amplification cycle

4.1 NMR observation of signal-amplification cycle with 5% signal

Compound **3** (1.91 mg, 8.10 μM, 1.5 eq.), acrylate-2 (3.49 mg, 3.0 eq.) and DSS internal standard (1.47 mg, 1.0 eq.) were mixed in 0.4 mL phosphate buffer (pH = 7.6) and shaken for 30 minutes. To this solution compound **1** (5.0 mg, 1.0 eq.) in 0.1 mL H_2O was added and the mixture was shaken for another 30 minutes before freeze dried. The remains were solubilized in 0.5 mL (H_2O/D_2O 8:2). The final solvent conditions were 0.5 mL D_2O/phosphate buffer 2:8, 0.1 M, pH = 7.6). The reaction was immediately followed by ^1H NMR spectroscopy to capture t = 0. At t = 10 minutes, compound **4** (0.04 mg, 0.05 eq.) was added to the NMR tube and the reaction was continuously measured at the appropriate time points over 24 hours.

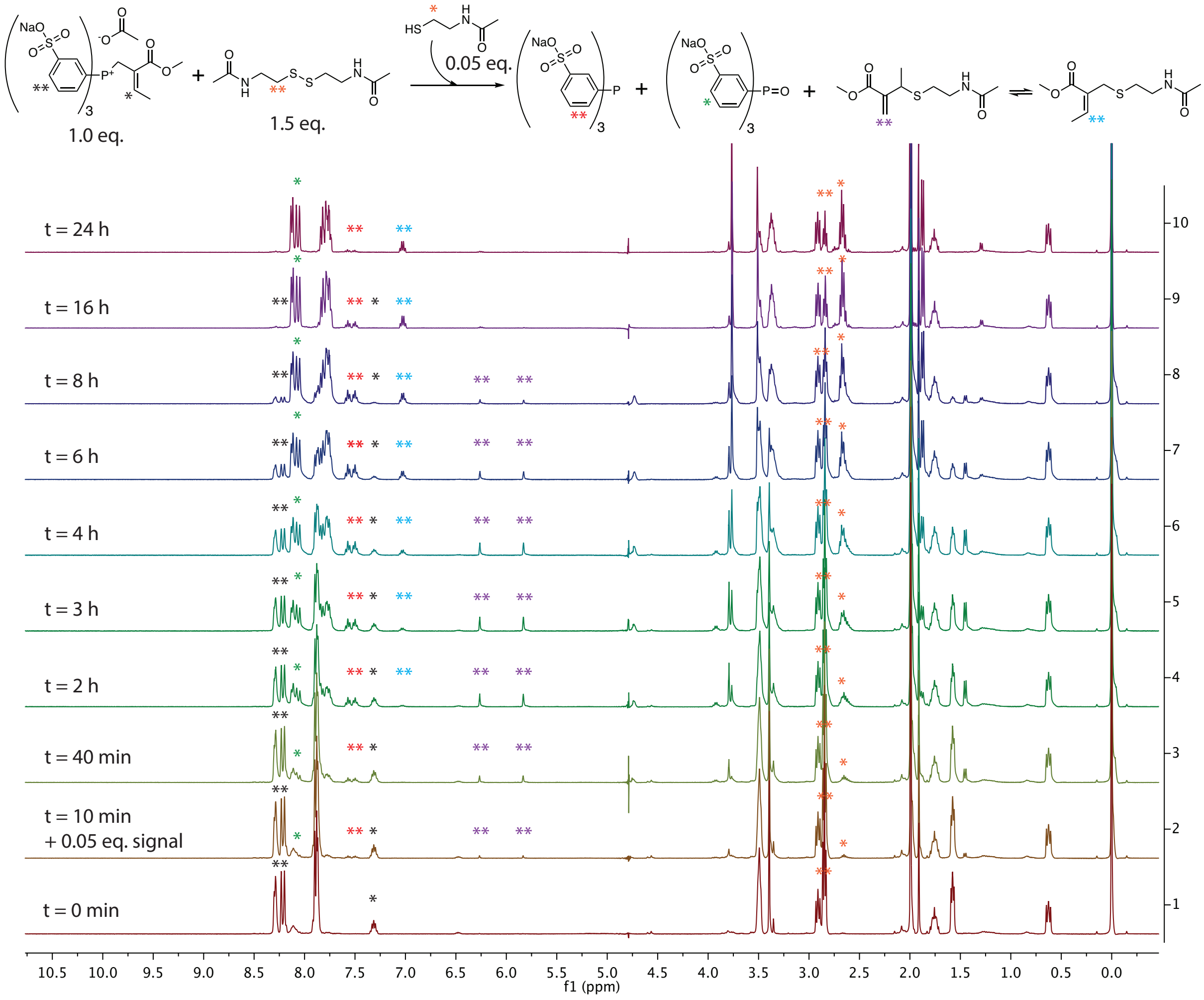

Supplementary Figure 11: Auto-amplification cycle observation with 5% SH-signal in ^1H NMR at different time points for 24 hours. The reaction was carried out in D_2O/phosphate buffer mixture 2:8 (0.1 M, pH = 7.6). The peak attributed to ~ 0.0 ppm corresponds to DSS internal standard and was used to align the spectra.

5.0 Kinetic model

A kinetic model for the signal-triggered amplification system was developed in PYTHON. In order to investigate and model the reaction system; forward (1) and backward (2) reaction pathways were separately performed, and experimental data for the reaction rates were collected. The rate constants for the forward and backward reaction were determined by UV-vis, following the appearance and disappearance of TPPTS at 260 and 300 nm, respectively.

5.1 UV-vis spectroscopy – rate constant determination

The nucleophilic substitution reaction (1), was performed at pseudo-first order regime - kinetics by using one of the reactants in excess. We performed kinetic experiments using 0.002 M (1.0 eq.) of **1** and exposed it to 0.0002 M (0.1 eq.) of **4**. All measurements were carried out in 0.1 M phosphate buffer (pH = 7.6) and at room temperature 25 °C. The pH was verified after the reaction was completed (~20 hours), and no change was observed.

**Nucleophilic substitution reaction (1)**	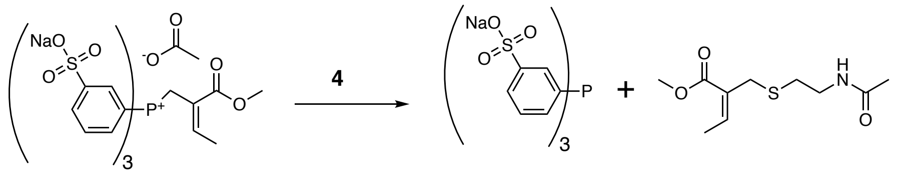 **1**	**TPPTS**	**2**
t = 0	$[A]_0$	$[A]_0$	0
t = t	$[A]_t = [A]_0 - [C]_t$	$[B]_t = [B]_0 - [C]_t$	$[C]_t$

$[A]_0$ is the excess compound **1** concentration, [B] the concentration of compound **4** and $[C]_t$ the product TPPTS concentration over time. The pseudo first-order regime - reaction rate constant was determined by fitting the production of TPPTS over time with the following equation:

$$ln\left[1-\frac{[C]_t}{[B]_0}\right] = -k_1[A]_0 t \qquad \text{Eq.3}$$

, where $[B]_0$ = initial concentration of **4** at t_0, 0.0002 M; $[C]_t$ = the concentration of TPPTS at every specified time obtained from UV-vis spectroscopy (Supplementary Figure 12a); k_1 is the rate constant (M^{-1} s^{-1}), $[A]_0$ = initial concentration of **1**, 0.002 M.

Similarly, for the disulfide reduction reaction (2), we used compound **3** in excess of 30 mM (1.0 eq.) and exposed it to 3.0 mM (0.1 eq.) of TPPTS. All measurements were carried out in 0.1M phosphate buffer (pH = 7.6) and at room temperature 25 °C. The pH was verified after the reaction was completed (~25 hours), and no change was observed.

Disulfide reduction reaction (2)

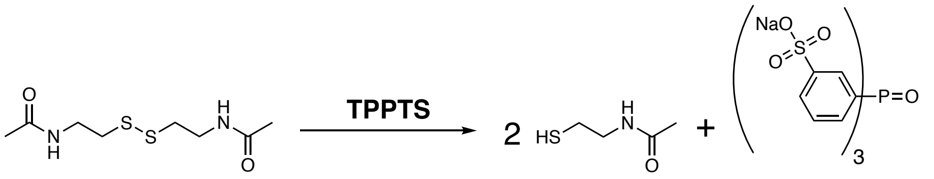

	3	4	OTPPTS
t = 0	$[A]_0$	$[A]_0$	0
t = t	$[A]_t = [A]_0 – [C]_t$	$[B]_t = [B]_0 – [C]_t$	$[C]_t$

$[A]_0$ is the excess compound 3 concentration, [B] the concentration of TPPTS and $[C]_t$ is the product OTPPTS concentration over time. The pseudo first-order reaction rate constant was determined by fitting the conversion of TPPTS over time with the following equation:

$$ln\left[\frac{[B]_t}{[B]_0}\right] = -k_2[A]_0 t \qquad \text{Eq.4}$$

, where $[B]_0$ = initial concentration of 3 at t_0, 0.030 M; $[B]_t$ = the concentration of TPPTS at every specified time point, obtained from UV-vis spectroscopy (Supplementary Figure 12b); k_2 is the reaction rate constant (M^{-1} s^{-1}), $[A]_0$ = initial concentration of TPPTS, 0.003 M.

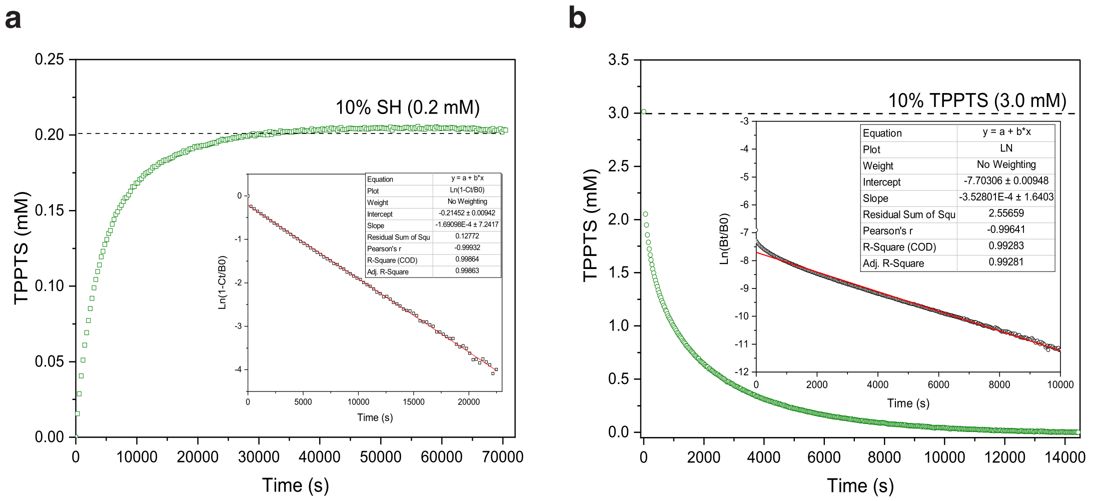

Supplementary Figure 12: TPPTS concentrations obtained by UV-vis measurement and calculated slopes for the (a) nucleophilic substitution and (b) disulfide reduction reaction. Conditions: (a) 0.002 M (1.0 eq.) of **1** and 0.0002 M (0.1 eq.) of **4**, (b) 0.030 M (1.0 eq.) of **3** and 0.003 M (0.1 eq.) of TPPTS in 0.1 M phosphate buffer (pH = 7.6) at 25 °C.

The linearity of $ln(1-[C_t]/[B_0])$ and $ln[B_t]/[B_0]$ versus time graphs gave the reaction rate constants of $k_1 = 8.450*10^{-2}$ $M^{-1}s^{-1}$ and $k_2 = 1.176*10^{-2}$ $M^{-1}s^{-1}$ for the nucleophilic substitution and disulfide reduction reaction, respectively.

5.2 Forward reaction: TPPTS release modelling

A simplified mathematical model was developed based on a set of linear differentials describing the nucleophilic substitution reaction of **1** with **4** and solved numerically for a series of reactions, which were compared to experimental measurements from UV-vis. To begin we developed a one-step reaction model, based on Scheme 1:

Supplementary Scheme 1: Forward reaction (nucleophilic substitution) of **1** with **4** for one-step model.

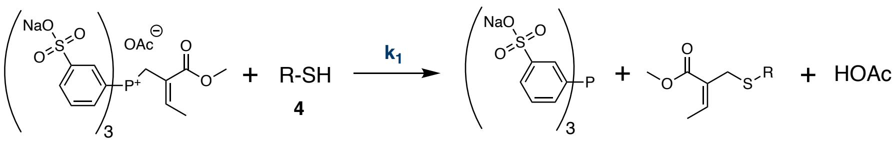

Rate equations of all compounds involved in the TPPTS release were established according to a one-step second order reaction model, as shown below:

$$\frac{d[TPPTS]}{dt} = +k_1.[1].[4] \quad \text{Eq. 5}$$

$$\frac{d[1]}{dt} = -k_1.[1].[4] \quad \text{Eq. 6}$$

$$\frac{d[4]}{dt} = -k_1.[1].[4] \quad \text{Eq. 7}$$

$$\frac{d[2]}{dt} = +k_1.[1].[4] \quad \text{Eq. 8}$$

$$\frac{d[HOAc]}{dt} = +k_1.[1].[4] \quad \text{Eq. 9}$$

This set of ordinary differential equations was then solved over the experimental timeframe. The rate constant measured in the pseudo-first-order regime (Section 4.1) was used to achieve the TPPTS concentration profiles at different signal levels (thiol concentration). Supplementary Figure 13, illustrates the comparison between model predictions and the measured TPPTS concentration progression. By using the experimentally determined k_1-value for the prediction of TPPTS release, we found that the model cannot predict satisfactory the experimental data for low concentrations of SH-input **4**.

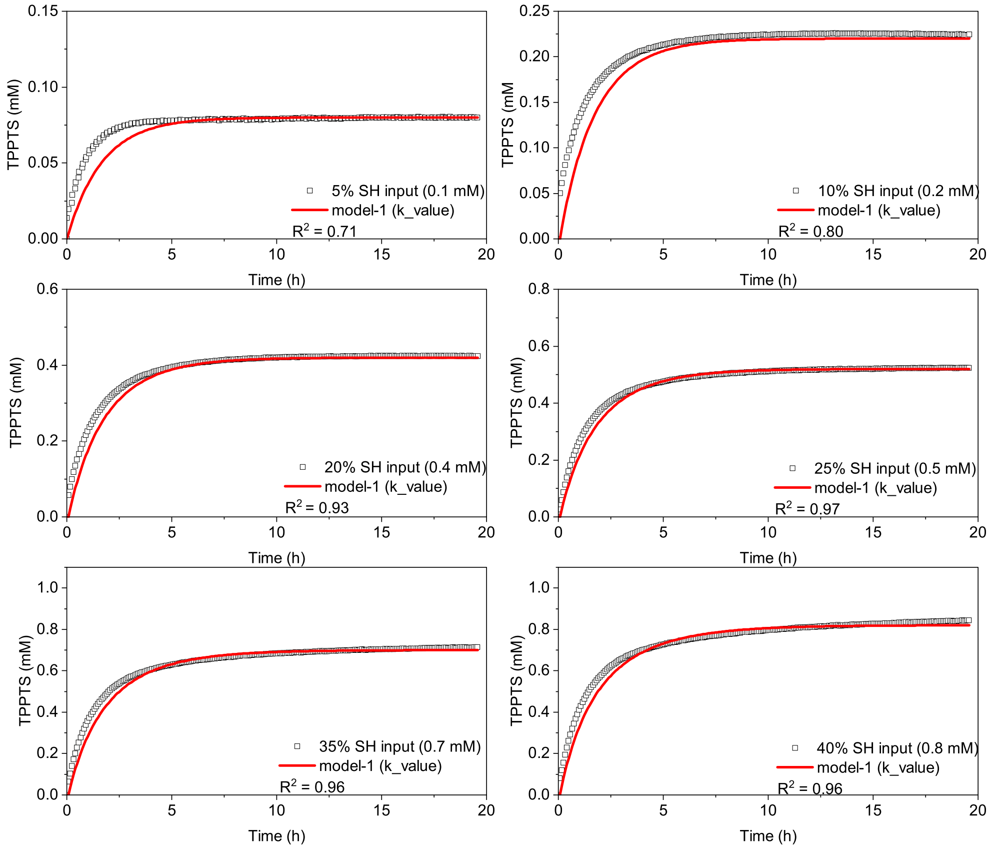

Supplementary Figure 13: TPPTS concentrations obtained by UV-vis measurement and model predictions (red line) with one-step reaction pathway using experimentally determined k_1-value for different concentrations of SH-signal input. Conditions: 0.002 M (1.0 eq.) of **1** and appropriate amounts of **4** in 0.1 M phosphate buffer (pH =7.6) at room temperature 25 °C. All experimental measurements were done in duplicate. R^2 values are shown as indicator for fitting between experimental measurements and model prediction.

After attempts at optimizing the rate constant, no single value was found to make the predictions match the experimental TPPTS concentration progression. Thus, inspired by previous work from Krische and coworkers[1], on the mechanism of this reaction a two-step reaction model was proposed, as shown in Supplementary Scheme 2:

Supplementary Scheme 2: Forward reaction (nucleophilic substitution) of **1** with **4** for two-step model.

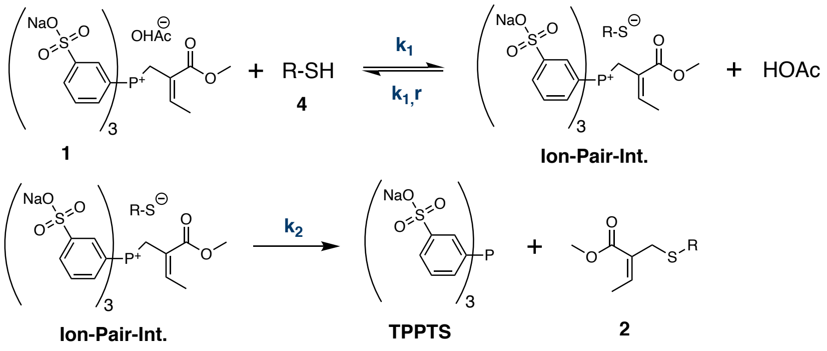

In this mechanism, a reversible acid/base reaction between (acetate) and (thiol), leads to the formation of an intermediate compound (Ion-Pair-Int.), which decomposes to TPPTS and **2**. Following, rate equations were developed according to this new model, as shown below:

$$\frac{d[TPPTS]}{dt} = +k_2.[Ion - Pair - Int.] \quad \text{Eq. 10}$$

$$\frac{d[1]}{dt} = -k_1.[1].[4] + k_{1r}.[Ion - Pair - Int.].[HOAc] \quad \text{Eq. 11}$$

$$\frac{d[4]}{dt} = -k_1.[1].[4] + k_{1r}.[Ion - Pair - Int.].[HOAc] \quad \text{Eq. 12}$$

$$\frac{d[2]}{dt} = +k_2.[Ion - Pair - Int.] \quad \text{Eq. 13}$$

$$\frac{d[Ion - Pair - Int.]}{dt} = +k_1.[1].[4] - k_{1r}.[Ion - Pair - Int.].[HOAc] - k_2.[Ion - Pair - Int.] \quad \text{Eq. 14}$$

$$\frac{d[HOAc]}{dt} = +k_1.[1].[4] - k_{1r}.[Ion - Pair - Int.].[HOAc] \quad \text{Eq. 15}$$

These new rate constants were then calculated by fitting the model predicted TPPTS concentration profile to the experimental data. By using least squares method, k_1, k_{1r} and k_2 were determined to be 0.1314 $M^{-1}s^{-1}$, 0.998 $M^{-1}s^{-1}$ and 0.0020 $M^{-1}s^{-1}$. Accuracy of the predicted TPPTS concentration profiles with the two-step model, using above rate constants, were measured at different initial concentrations. By using the optimized k-values for the prediction of TPPTS release, we found good agreement between model and the experimental data for a variety of SH-input **4** (Supplementary Figure 14).

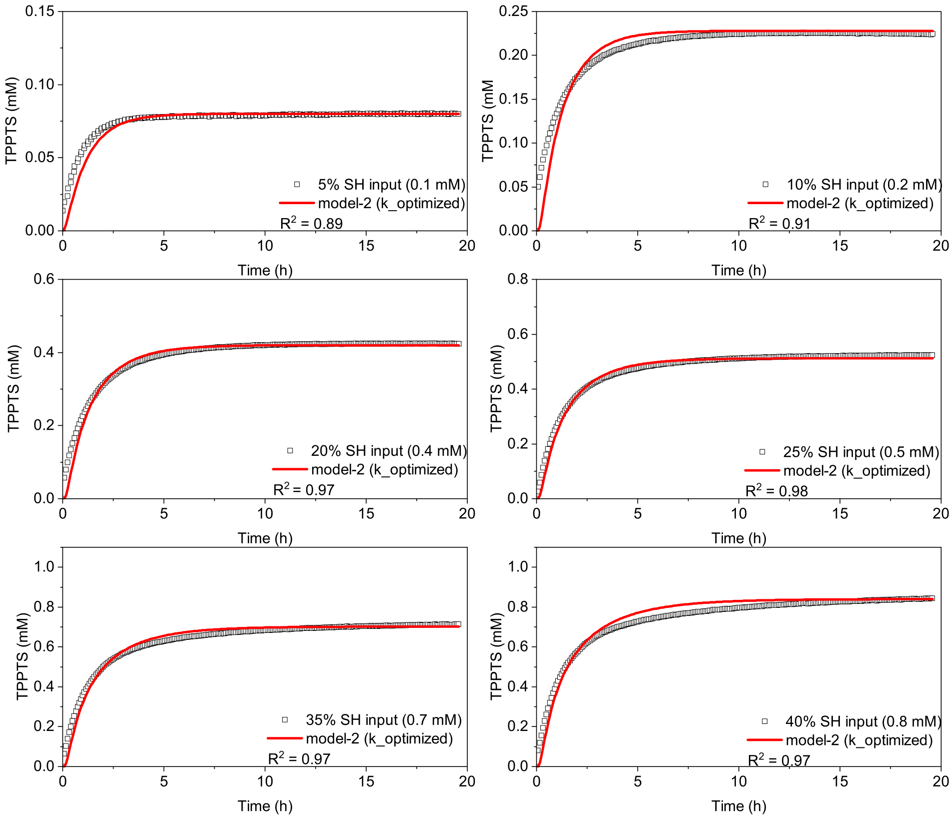

Supplementary Figure 14: TPPTS concentrations obtained by UV-vis measurement and model predictions (red line) with two-step reaction pathway using the optimized k-values for different concentrations of SH-signal input. Conditions: 0.002 M (1.0 eq.) of **1** and appropriate amounts of **4** in 0.1 M phosphate buffer (pH = 7.6) at room temperature 25 °C. All experimental measurements were done in duplicate. R^2 values are shown as indicator for fitting between experimental measurements and model prediction.

5.3 Backward reaction: disulfide reduction modelling

A simplified mathematical model was developed based on a set of linear differentials describing the disulfide reduction reaction of **3** with TPPTS and solved numerically for a series of reactions, which were compared to experimental measurements from UV-vis. To begin we developed a one-step reaction model, based on Supplementary Scheme 3:

Supplementary Scheme 3: Backward reaction (disulfide reduction) of **3** with TPPTS for one-step model.

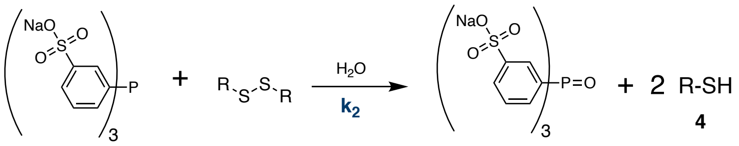

TPPTS **3** **OTPPTS**

Rate equations for all of the species in the disulfide reduction, based on a one-way second order reaction rate, have been developed as following:

$$\frac{d[TPPTS]}{dt} = -k_2.[TPPTS].[3] \quad \text{Eq. 16}$$

$$\frac{d[3]}{dt} = -k_2.[TPPTS].[3] \quad \text{Eq. 17}$$

$$\frac{d[OTPPTS]}{dt} = k_2.[TPPTS].[3] \quad \text{Eq. 18}$$

$$\frac{d[4]}{dt} = 2.k_2.[TPPTS].[3] \quad \text{Eq. 19}$$

By using the rate constant attained through pseudo-first order analysis (Section 4.1), the aforementioned system of ordinary differential equations was solved with the initial concentrations, resulting in concentration profiles for all the species over the experimental timeframe. Supplementary Figure 15, illustrates the comparison between the predictions and actual TPPTS concentration profiles at different initial conditions. We found that the model cannot predict satisfactory the experimental data for all concentrations of TPPTS.

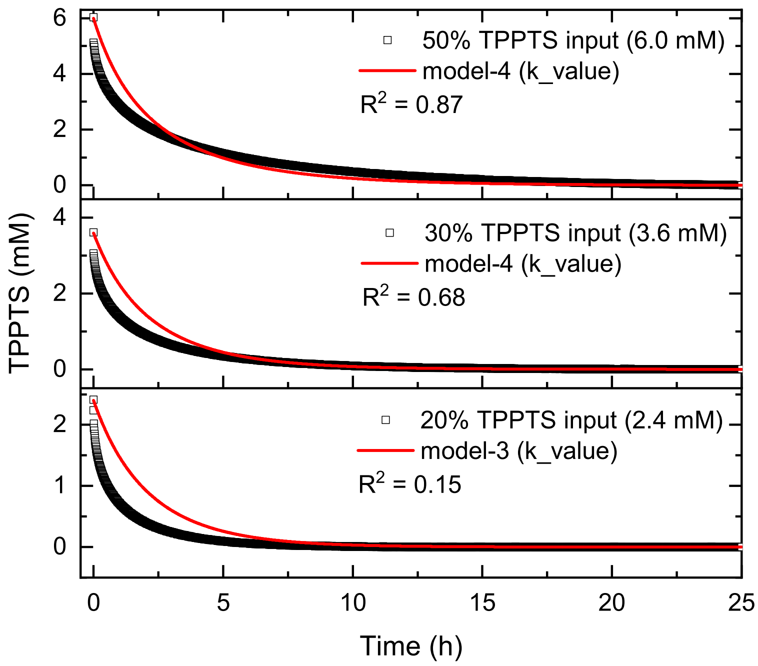

Supplementary Figure 15: TPPTS concentrations obtained by UV-vis measurement and model predictions (red line) with one-step reaction pathway using the experimentally determined k_2-value for different concentrations of TPPTS input. Conditions: 0.012 M (1.0 eq.) of **3** and appropriate amounts of TPPTS in 0.1 M phosphate buffer (pH = 7.6) at 25 °C. All experimental measurements were done in duplicate. R^2 values are shown as indicator for fitting between experimental measurements and model prediction.

Simply optimizing the rate constant did not significantly reduce the discrepancy between the predictions and the actual data. Instead, a two-step model was proposed as described by Bach and coworkers.[2], forming a phosphine-thiol intermediate (PR_3^+-thiol-Int.) and releasing one thiol **4** within each step, according to Supplementary Scheme 4:

Supplementary Scheme 4: Backward reaction (disulfide reduction) of **3** with TPPTS for two-step model.

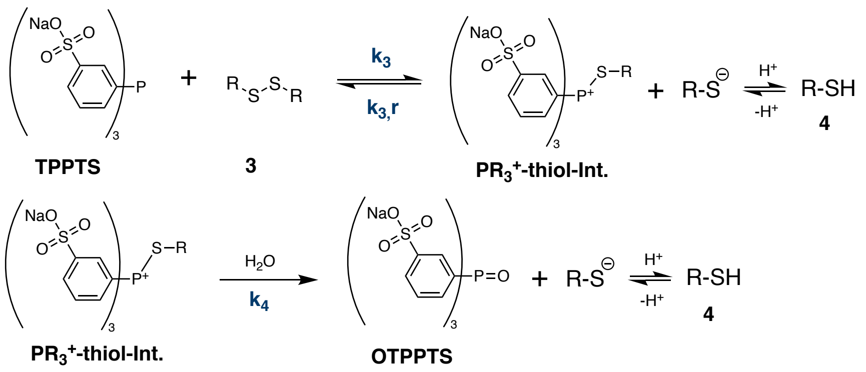

Following rate equations were developed according to this new model, as shown below:

$$\frac{d[TPPTS]}{dt} = -k_3.[TPPTS].[3] + k_{3r}.[PR_3^+ - thiol - int.].[4] \quad \text{Eq. 20}$$

$$\frac{d[3]}{dt} = -k_3.[TPPTS].[3] + k_{3r}.[PR_3^+ - thiol - int.].[4] \quad \text{Eq. 21}$$

$$\frac{d[OTPPTS]}{dt} = k_4.[PR_3^+ - thiol - int.] \quad \text{Eq. 22}$$

$$\frac{d[4]}{dt} = k_3.[TPPTS].[3] - k_{3r}.[PR_3^+ - thiol - int.].[4] + k_4.[PR_3^+ - thiol - int.] \quad \text{Eq. 23}$$

$$\frac{d[PR_3^+ - thiol - int.]}{dt} = k_3.[TPPTS].[3] - k_{3r}.[PR_3^+ - thiol - int.].[4] - k_4.[PR_3^+ - thiol - int.] \quad \text{Eq. 24}$$

The TPPTS concentration profile, predicted by the new model, was fitted to the experimental findings and through least squares method the rate constants were achieved. k_3, k_{3r} and k_4 are 9.3*10^{-2} $M^{-1}s^{-1}$, 0.74 $M^{-1}s^{-1}$ and 7.1*10^{-4} $M^{-1}s^{-1}$, respectively. These values were consistent over changes in initial conditions. Supplementary Figure 16, compares the predictions with the optimized rate constants to the actual experimental data. By using the optimized *k*-values for the prediction of TPPTS conversion, we found excellent agreement between model and the experimental data (Supplementary Figure 16).

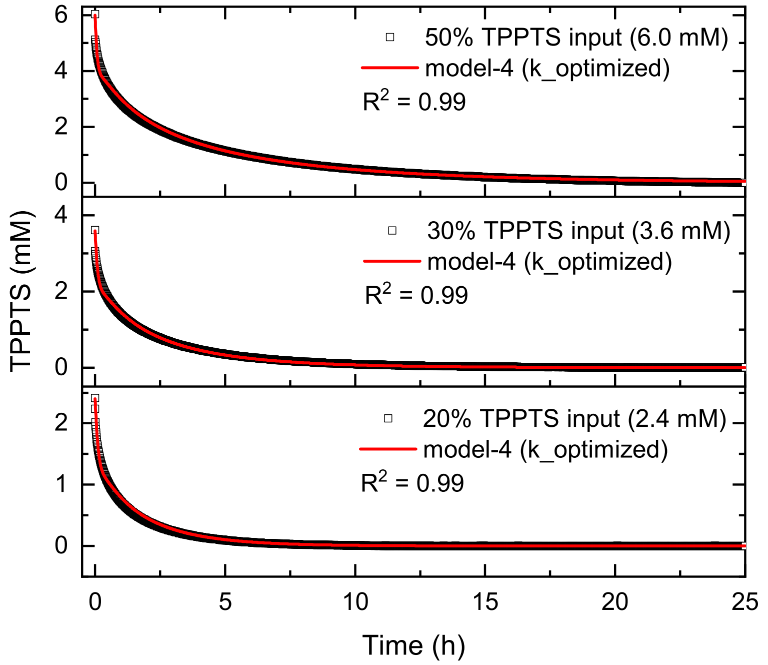

Supplementary Figure 16: TPPTS concentrations obtained by UV-vis measurement and model predictions (red line) with two-step reaction pathway using the optimized *k*-values for different concentrations of TPPTS input. Conditions: 0.012 M (1.0 eq.) of **3** and appropriate amounts of TPPTS in 0.1 M phosphate buffer (pH = 7.6) at 25 °C. All experimental measurements were done in duplicate. R^2 values are shown as indicator for fitting between experimental measurements and model prediction.

5.4 Signal-amplification cycle - model

A model was proposed for the whole reaction cycle which encompasses the combined rate equations developed for the forward and the backward reaction pathways, as shown below:

$$\frac{d[TPPTS]}{dt} = +k_2.[Ion - Pair - Int.] - k_3.[TPPTS].[3] + k_{3r}.[PR_3^+ - thiol - int.].[4] \quad \text{Eq. 25}$$

$$\frac{d[1]}{dt} = -k_1.[1].[4] + k_{1r}.[Ion - Pair - Int.].[HOAc] \quad \text{Eq. 26}$$

$$\frac{d[4]}{dt} = -k_1.[1].[4] + k_{1r}.[Ion - Pair - Int.].[HOAc]k_3.[TPPTS].[3] - k_{3r}.[PR_3^+ - thiol - int.].[4] + k_4.[PR_3^+ - thiol - int.] \quad \text{Eq. 27}$$

$$\frac{d[2]}{dt} = +k_2.[Ion - Pair - Int.] \quad \text{Eq. 28}$$

$$\frac{d[Ion - Pair - Int.]}{dt} = +k_1.[1].[4] - k_{1r}.[Ion - Pair - Int.].[HOAc] - k_2.[Ion - Pair - Int.] \quad \text{Eq. 29}$$

$$\frac{d[HOAc]}{dt} = +k_1.[1].[4] - k_{1r}.[Ion - Pair - Int.].[HOAc] \quad \text{Eq. 30}$$

$$\frac{d[3]}{dt} = -k_3.[TPPTS].[3] + k_{3r}.[PR_3^+ - thiol - int.].[4] \quad \text{Eq. 31}$$

$$\frac{d[OTPPTS]}{dt} = k_4.[PR_3^+ - thiol - int.] \quad \text{Eq. 32}$$

$$\frac{d[PR_3^+ - thiol - int.]}{dt} = k_3.[TPPTS].[3] - k_{3r}.[PR_3^+ - thiol - int.].[4] - k_4.[PR_3^+ - thiol - int.] \quad \text{Eq. 33}$$

6.0 Hydrogels – signal-amplification experiments

6.1 Photo-observation of signal-amplified hydrogel degradation study

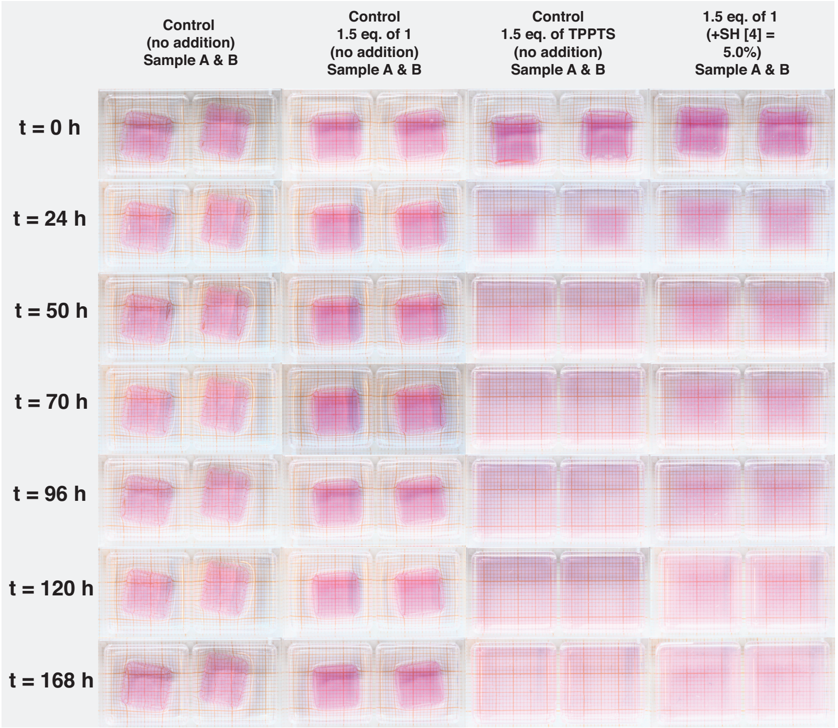

Supplementary Figure 17: Time lapse photographs (in duplicate) of hydrogel degradation using the self-amplification system triggered by SH-analytes. (a) Control gels with 1) no additives, 2) with 1.5 eq. of **1**, 3) 1.5 eq. of TPPTS and 4) 1.5 eq. of **1** and 5% (0.05 eq.) of **4**. Conditions: gels were submerged in 1.5 mL phosphate buffer (0.1 M, pH = 7.6).

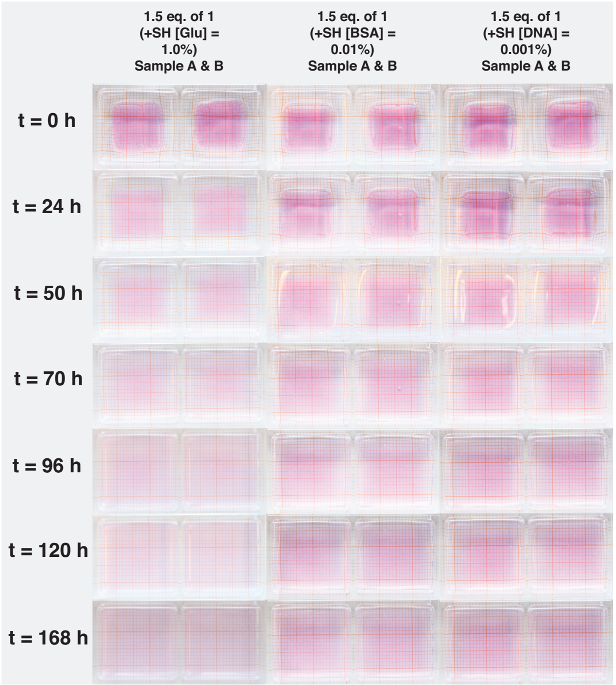

Supplementary Figure 18: Time lapse photographs (in duplicate) of hydrogel degradation using the self-amplification system triggered by SH-analytes. Gels with 1.5 eq. of **1** and SH-trigger addition of 1) 1.0% (0.01 eq.) of glutathione, 2) 0.01% (0.0001 eq.) of bovine serum albumin and 3) 0.001% (0.00001 eq.) of thiol functionalized DNA. Conditions: gels were submerged in 1.5 mL phosphate buffer (0.1 M, pH = 7.6).

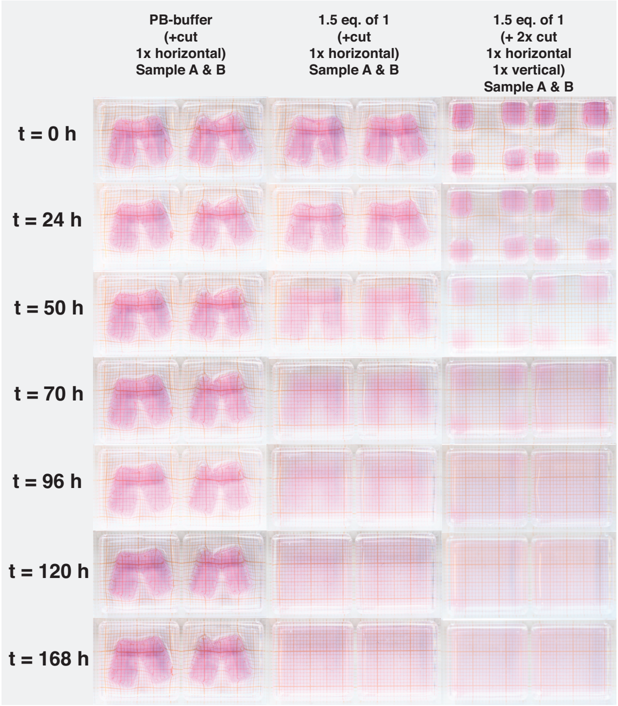

Supplementary Figure 19: Time lapse photographs (in duplicate) of hydrogel degradation using the self-amplification system triggered by damage (cut). (a) Control gels with 1) no additives and 1x horizontal cut, 2) 1.5 eq. of 1 and 1x horizontal cut, 3) 1.5 eq. of 1 and 2x cut (1x horizontal and 1x vertical). Conditions: gels were submerged in 1.5 mL phosphate buffer (0.1 M, pH = 7.6).

7.0 Synthesis of compounds

7.1 Synthesis of trisodium 3,3',3''-((2-(methoxycarbonyl)but-2-en-1-yl)phosphoniotriyl)tribenzenesulfonate) acetate (1)

Acrylate-1 (0.26 mmol, 43.9 mg, 1.0 eq.) and trisodium tris(3-sulfophenyl)phosphine (TPPTS) (0.16 mmol, 92.3 mg, 0.64 eq.) are dissolved in H_2O (1.0 mL) and stirred for 1 hour. The solution is then freeze dried to give the title compound (0.16 mmol, 116.8 mg, 97%) as white solid as an inseparable mixture of (E/Z) isomers in a 92:8 ratio (based on ^{1}H NMR).

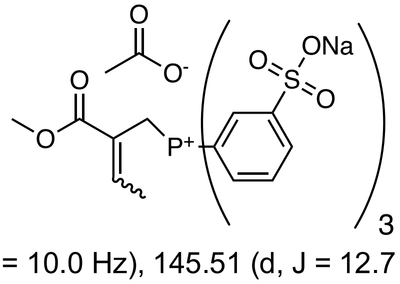

^{1}H NMR (400 MHz, D_2O + 1 µL DMSO, for E-isomer) δ: 8.21 – 8.06 (m, 6H), 7.74 (t, J = 5.8 Hz, 7H), 7.18 (p, J = 6.3 Hz, 1H), 4.45 (d, J = 14.4 Hz, 2H), 3.26 (s, 3H), 1.77 (s, 3H), 1.44 (t, J = 6.3 Hz, 3H). **^{13}C NMR** (101 MHz, D_2O + 1 µL DMSO, for E-isomer) δ: 182.26, 168.26 (d, J = 1.8 Hz), 150.07 (d, J = 10.0 Hz), 145.51 (d, J = 12.7 Hz), 137.84 (d, J = 10.2 Hz), 133.33 (d, J = 2.8 Hz), 132.06 (d, J = 12.6 Hz), 131.57 (d, J = 11.4 Hz), 120.87 (d, J = 9.9 Hz), 118.44 (d, J = 86.2 Hz), 53.39, 24.04, 23.60, 15.78 (d, J = 2.8 Hz). **^{31}P NMR** (161.9 MHz, D_2O + 1 µL DMSO, for E-isomer) δ: 20.79. **IR (KBr):** 3066, 2955 (C-H), 1715 (C=O), 1644 (C=C), 1581, 1407, 1398 (S=O), 1291, 1209, 1199 (S=O), 1102, 1039 (S=O), 993, 931, 843, 800, 792 (H-Ph), 727.

7.2 Synthesis of methyl 2-(((2-acetamidoethyl)thio)methyl)but-2-enoate (2)

Acrylate-1 (0.17 mmol, 30 mg, 1.0 eq.) and compound **4** (0.16 mmol, 19 mg, 0.91 eq.) were mixed in 1 mL buffer (pH ~8.0). After 48 hours the solution was extracted with ethyl acetate. The organic fraction was dried with Na_2SO_4, filtered and concentrated under reduced pressure to give compound 2 as colourless oil (0.136 mmol, 31.5 mg, 86%) as an inseparable mixture of (E/Z) isomers in a 80:20 ratio (based on ^{1}H NMR).

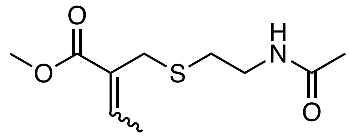

^{1}H NMR (400 MHz, $CDCl_3$, for E-isomer) δ: 6.94 (q, J = 7.2 Hz, 1H), 6.25 (s, 1H), 3.74 (s, 3H), 3.46 (q, J = 7.4 Hz, 2H), 3.45 (s, 2H), 2.64 (t, 2H), 1.98 (s, 3H), 1.86 (d, J = 7.2 Hz, 3H). **^{13}C NMR** (101 MHz, $CDCl_3$, for E-isomer) δ: 170.31, 167.33, 140.27, 129.83, 52.00, 38.64, 32.03, 26.76, 23.16, 14.64. **MS** (ESI+) m/z: 232.00 (M+H) (expected: 232.09).

7.3 Synthesis of N,N-diacetylcystamine (3)

N,N-diacetylcystamine was prepared as described elsewhere[3] with slight modifications. Briefly, a mixture of cysteamine dihydrochloride (10 mmol, 1.0 g, 1.0 eq.), KOH (20 mmol, 1.1 g, 2.0 eq.) and $NaHCO_3$ (30 mmol, 2.5 g, 3.0 eq.) were dissolved in a round-bottom flask containing 10 mL H_2O. After the dropwise addition of acetic anhydride (10 mmol, 1.0 g, 1.0

eq.), the solution was stirred at room temperature for 10 min. The pH was then adjusted to 7.3 using 4.0 M HCl. The resulting mixture was then extraction with 50 mL ethyl acetate three times and washed with brine. The organic layers were then dried with Na_2SO_4, filtered and concentrated under reduced pressure. After drying, the title compound was recrystalized 2x times in ethyl acetate, giving a white crystalline solid (2.4 mmol, 567 mg, 54%).

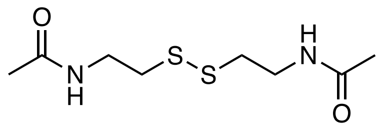

^{1}H NMR (400 MHz, DMSO-d6) δ: 8.03 (s, 2H), 3.30 (q, J = 6.6 Hz, 4H), 2.75 (t, J = 6.8 Hz, 4H), 1.80 (s, 6H). **^{13}C NMR** (101 MHz, DMSO-d6) δ: 169.31, 37.94, 37.25, 22.55.

7.4 Synthesis of N,N- Bis (acryloyl) cystamine (BAC)

BAC was prepared as described elsewhere[4]. Briefly, a mixture of cystamine dihydrochloride (6.9 mmol, 1.5 g, 1.0 eq.) was dissolved in a round-bottom flask containing 7.0 mL H_2O and cooled to 0 ºC. Two syringes filled with 1) acryloyl chloride solution (20.6 mmol, 1.9 g, 3.0 eq.) in 2 mL DCM and 2) NaOH (27.4 mmol, 1.1 g, 4.0 eq.) in 3 mL H_2O were simultaneously dropwise added to the solution and stirred for 16 hours at room temperature. After freeze drying, BAC was purified by recrystallization from ethyl acetate (3.57 mmol, 931 mg, 52%).

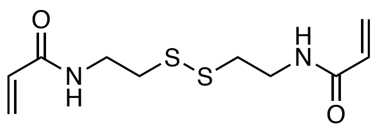

^{1}H NMR (400 MHz, DMSO-d6) δ: 8.30 (s, 1H), 6.22 (dd, J = 17.1, 10.1 Hz, 0H), 6.09 (d, J = 17.0 Hz, 0H), 5.59 (d, J = 10.2 Hz, 0H), 3.42 (q, J = 6.4 Hz, 2H), 2.82 (t, J = 6.8 Hz, 2H). **^{13}C NMR** (101 MHz, DMSO-d6) δ: 164.73, 131.55, 125.33, 37.93, 37.08.

7.5 Synthesis of tris(sodium-m-sulfonatophenyl)phosphanoxide (OTPPTS)

OTPPTS was prepared as described elsewhere[5]. Briefly, TPPTS (0.065 mmol, 37 mg) is dissolved in 1.0 mL of hydrogen peroxide (10% in water) and then the mixture is stirred overnight. Hereafter, the solution is freeze dried, giving OTPPTS as a white solid (0.063 mmol, 37 mg, 97%).

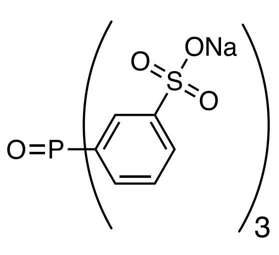

^{1}H NMR (400 MHz, D_2O + 5 µL DMSO) δ: 8.09 – 7.71 (m, 6H), 7.58 (dtd, J = 15.5, 7.7, 5.6 Hz, 6H). **^{13}C NMR** (101 MHz, D_2O + 5 µL DMSO) δ: 144.48, 144.36, 135.71, 135.60, 131.21, 131.19, 131.03, 130.90, 130.17, 129.49, 129.37. **^{31}P NMR** (161.9 MHz, D_2O + 5 µL DMSO) δ: 34.26.

8.0 NMR Spectra

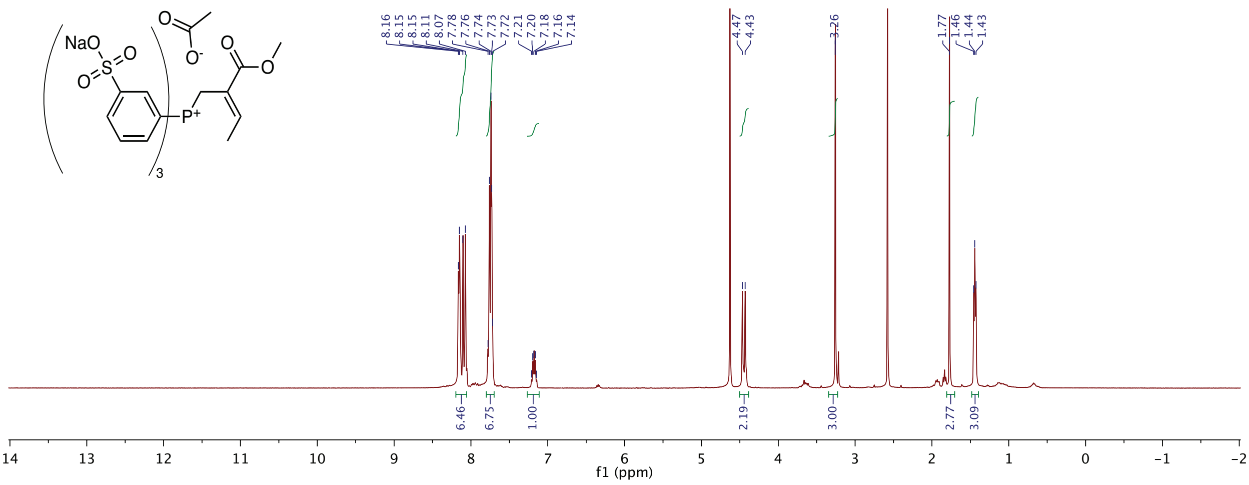

Supplementary Figure 20: ^{1}H NMR, compound **1** in D_2O + 1 µL DMSO.

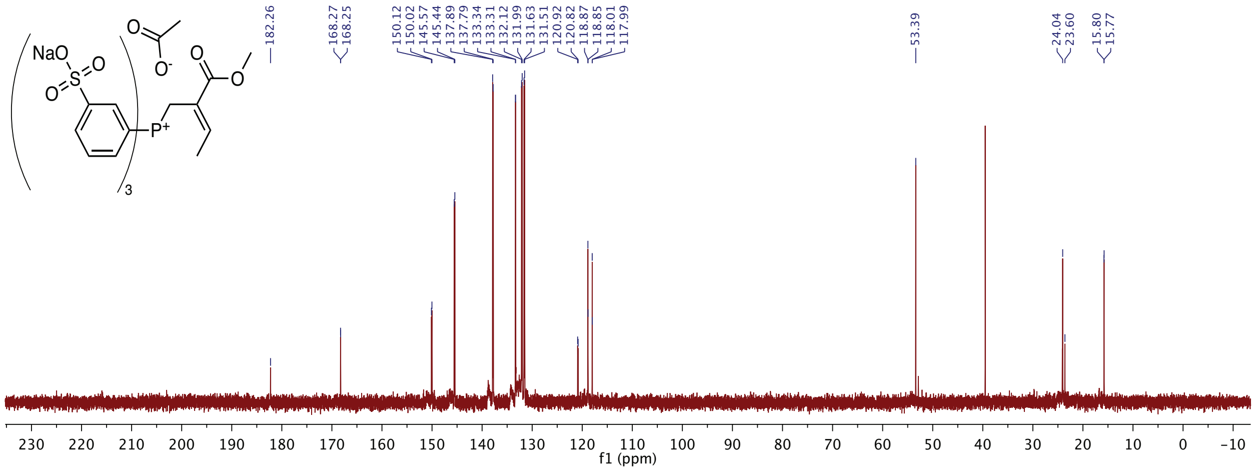

Supplementary Figure 21: ^{13}C NMR, compound **1** in D_2O + 1 µL DMSO.

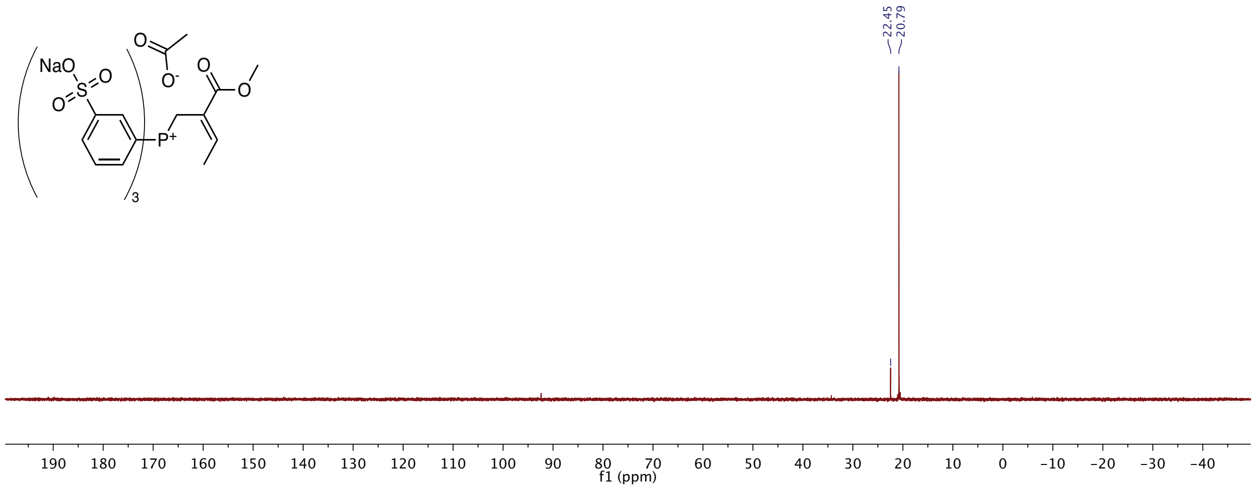

Supplementary Figure 22: ^{31}P NMR, compound **1** in D_2O + 1 µL DMSO.

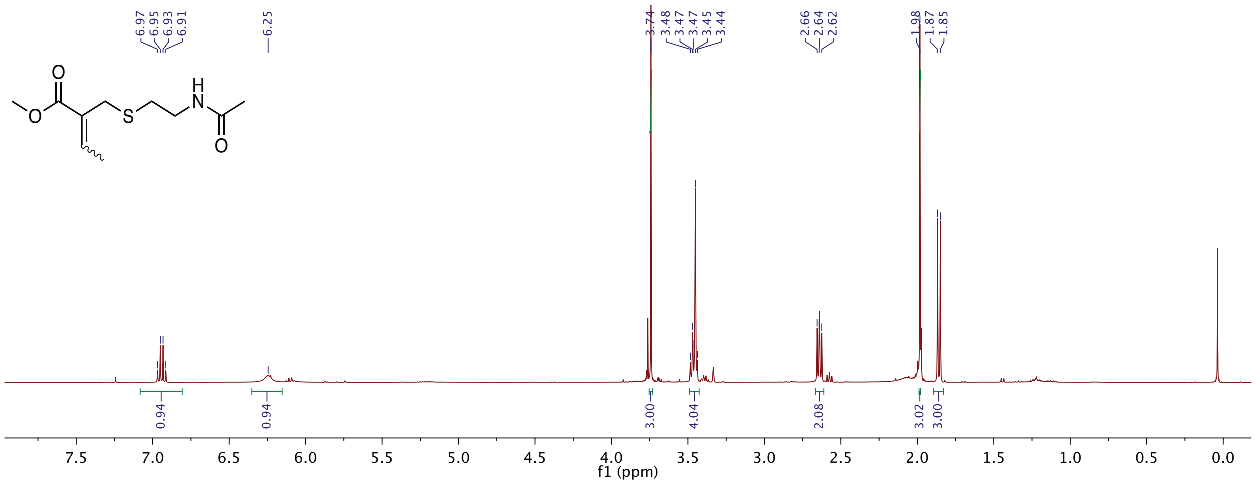

Supplementary Figure 23: ^{1}H NMR, compound **2** in $CDCl_3$.

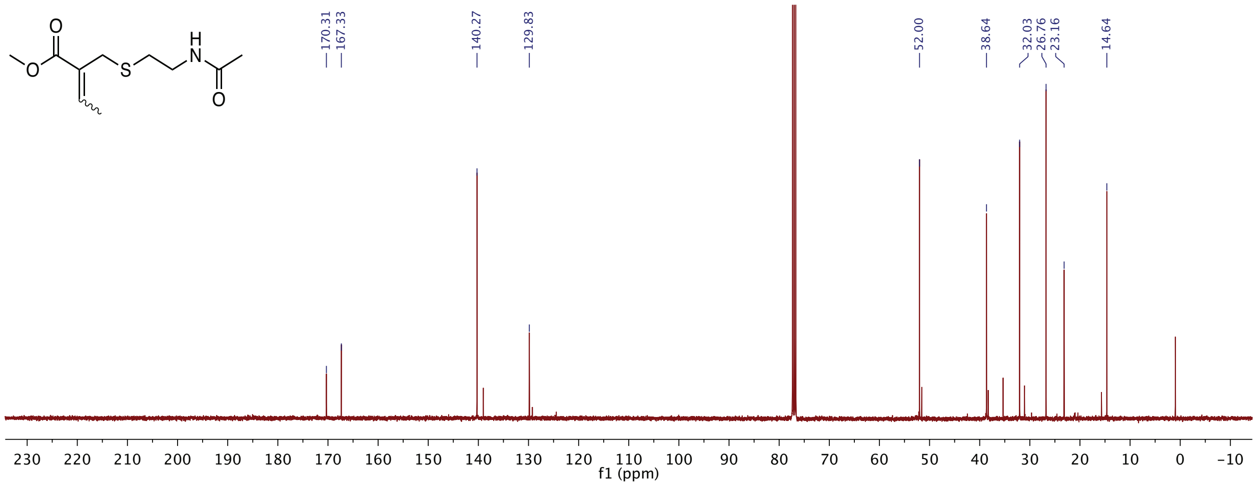

Supplementary Figure 24: ^{13}C NMR, compound **2** in $CDCl_3$.

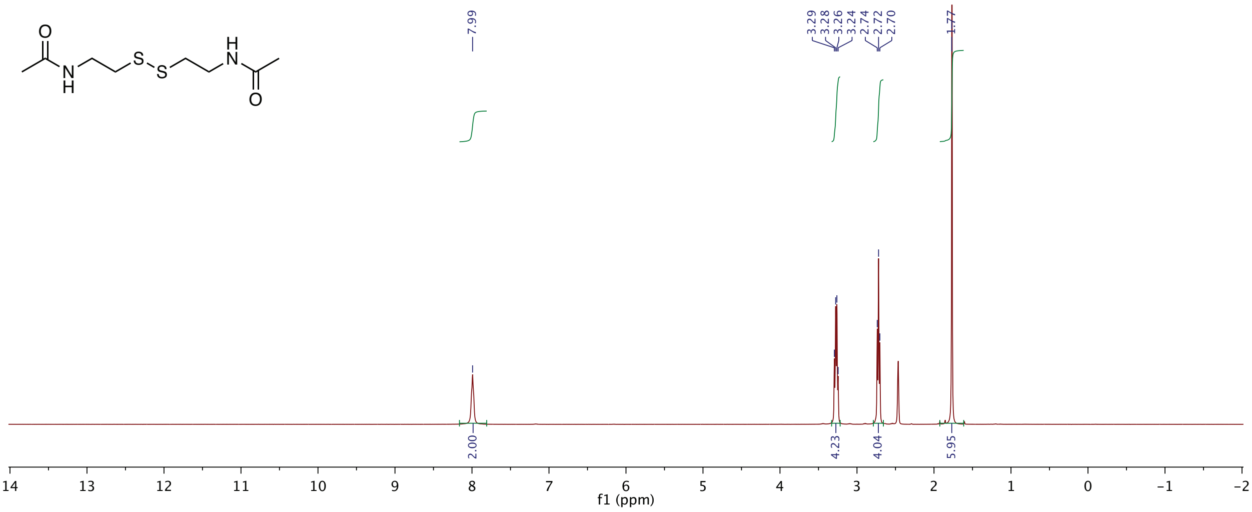

Supplementary Figure 25: ^{1}H NMR, compound **3** in DMSO-d6.

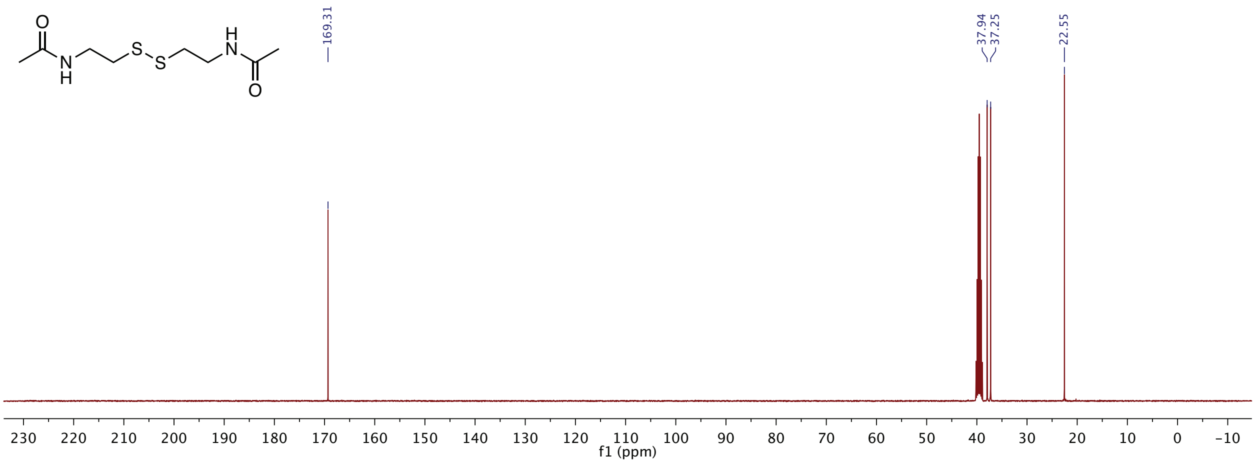

Supplementary Figure 26: ^{13}C NMR, compound **3** in DMSO-d6.

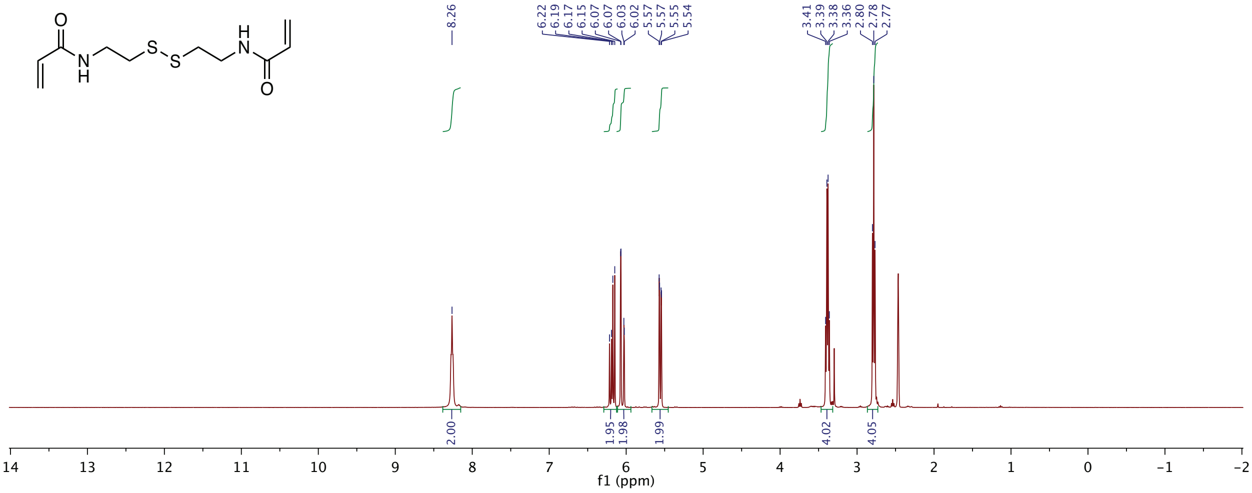

Supplementary Figure 27: ^{1}H NMR, compound BAC in DMSO-d6.

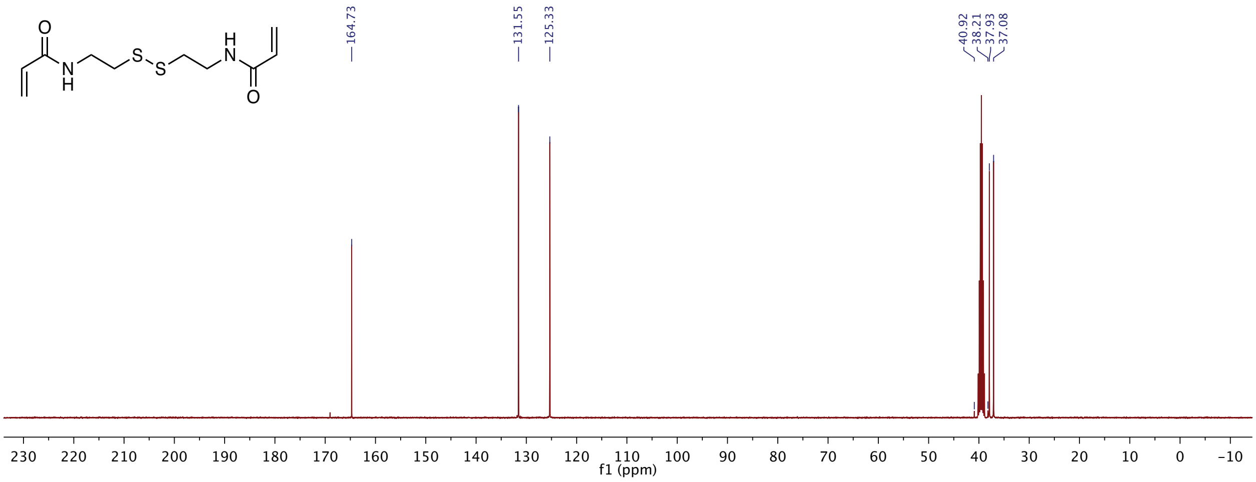

Supplementary Figure 28: ^{13}C NMR, compound BAC in DMSO-d6.

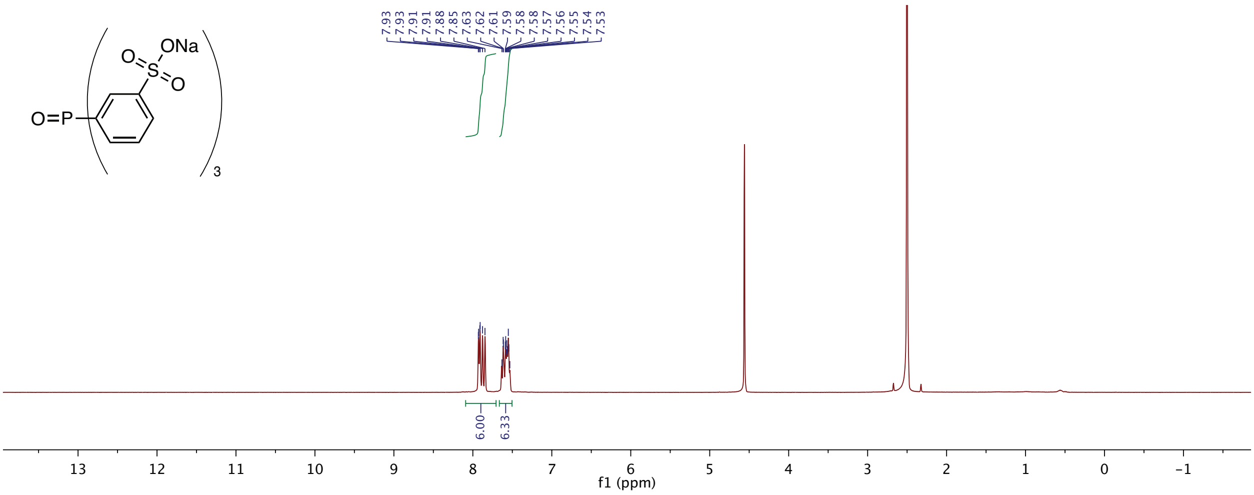

Supplementary Figure 29: ^{1}H NMR, compound OTPPTS in D_2O + 5 μL DMSO.

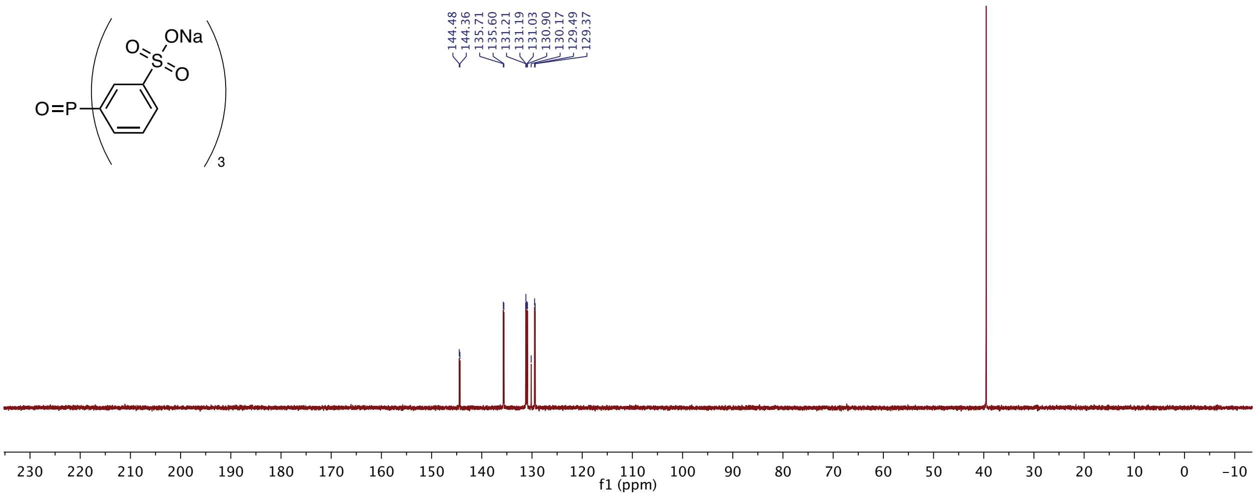

Supplementary Figure 30: ^{13}C NMR, compound OTPPTS in D_2O + 5 μL DMSO.

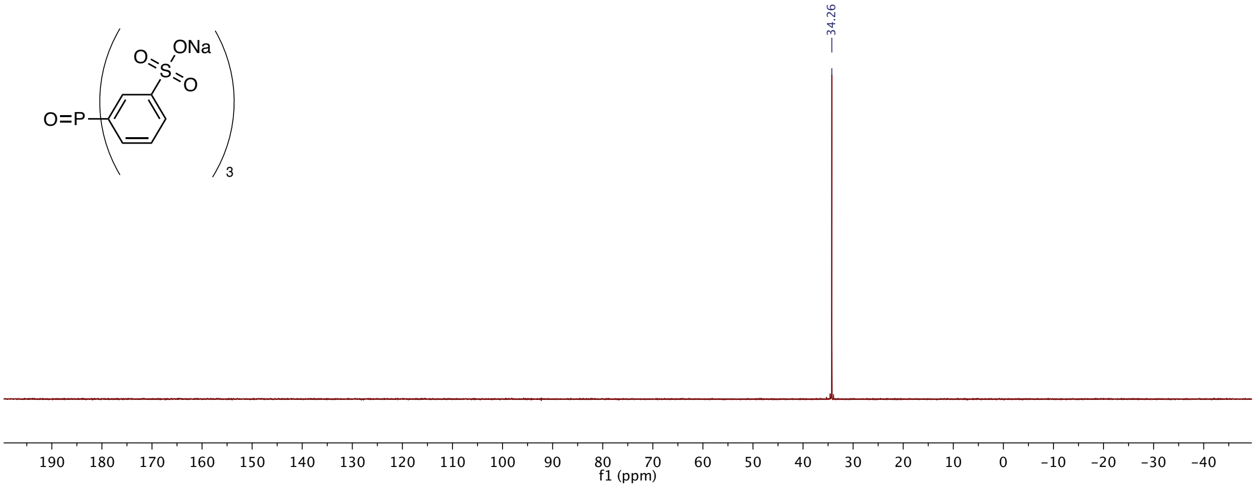

Supplementary Figure 31: ^{31}P NMR, compound OTPPTS in D_2O + 5 μL DMSO.

9.0 2-D NMR Spectra

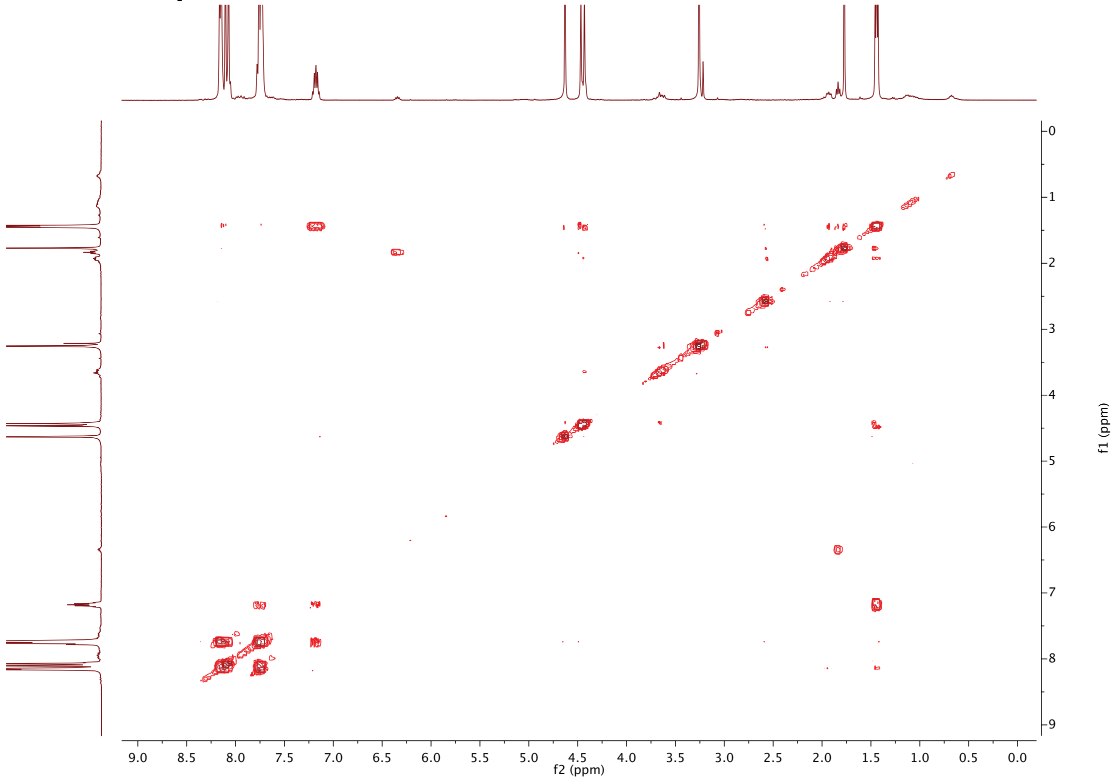

Supplementary Figure 32: gCOSY, compound **1** in D_2O + 1 µL DMSO.

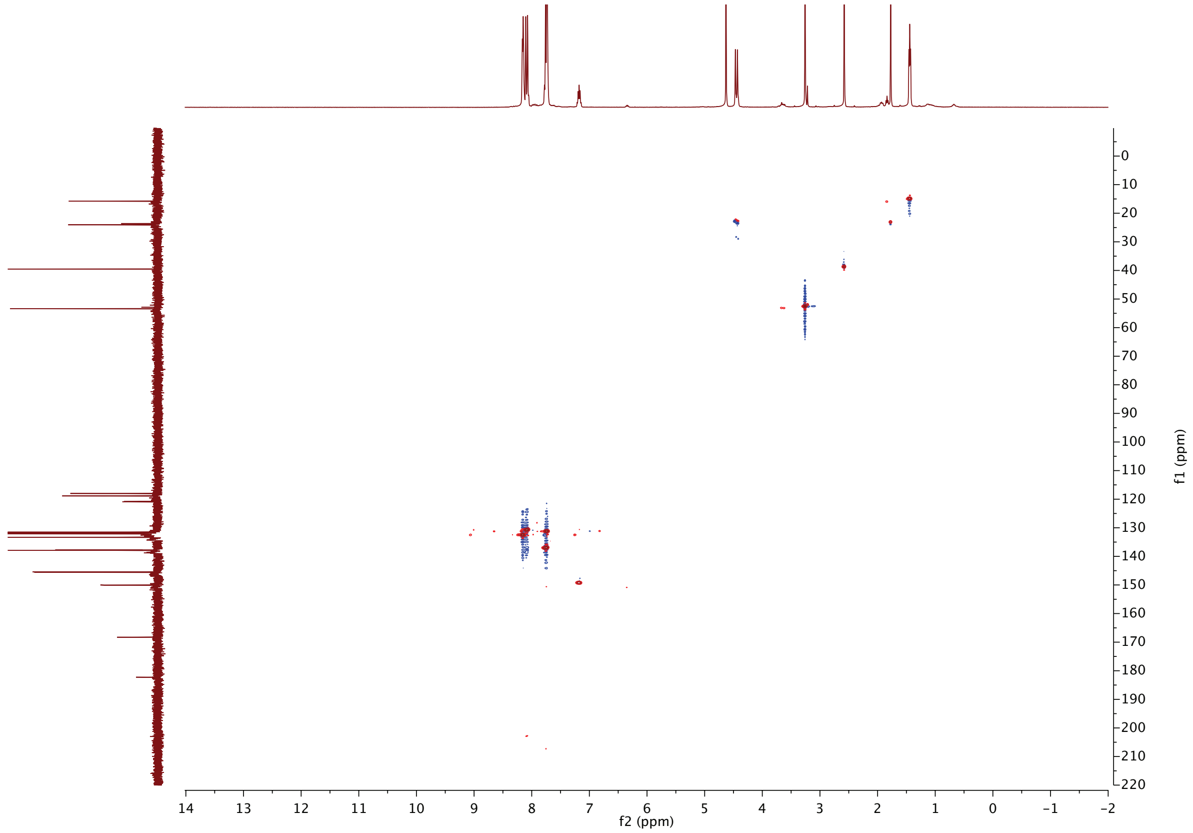

Supplementary Figure 33: gHSQC, compound **1** in D_2O + 1 µL DMSO.

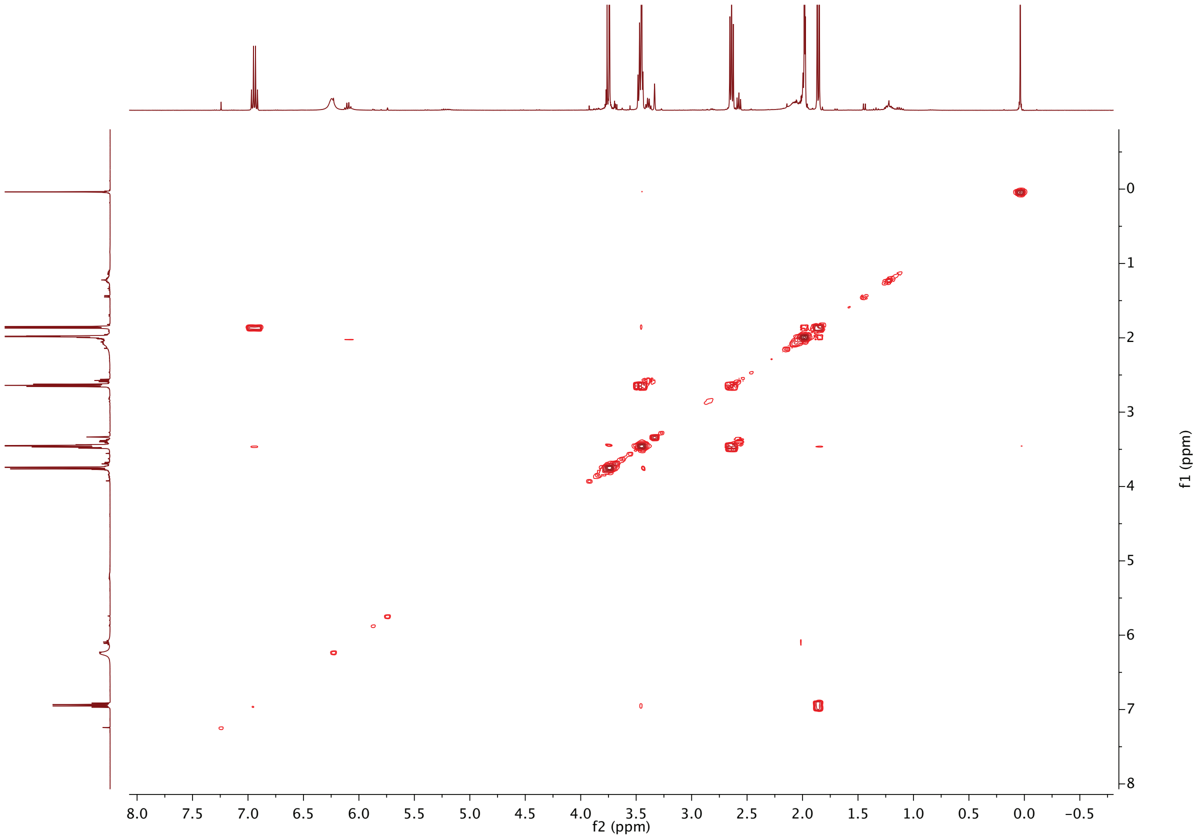

Supplementary Figure 34: gCOSY, compound **2** in $CDCl_3$.

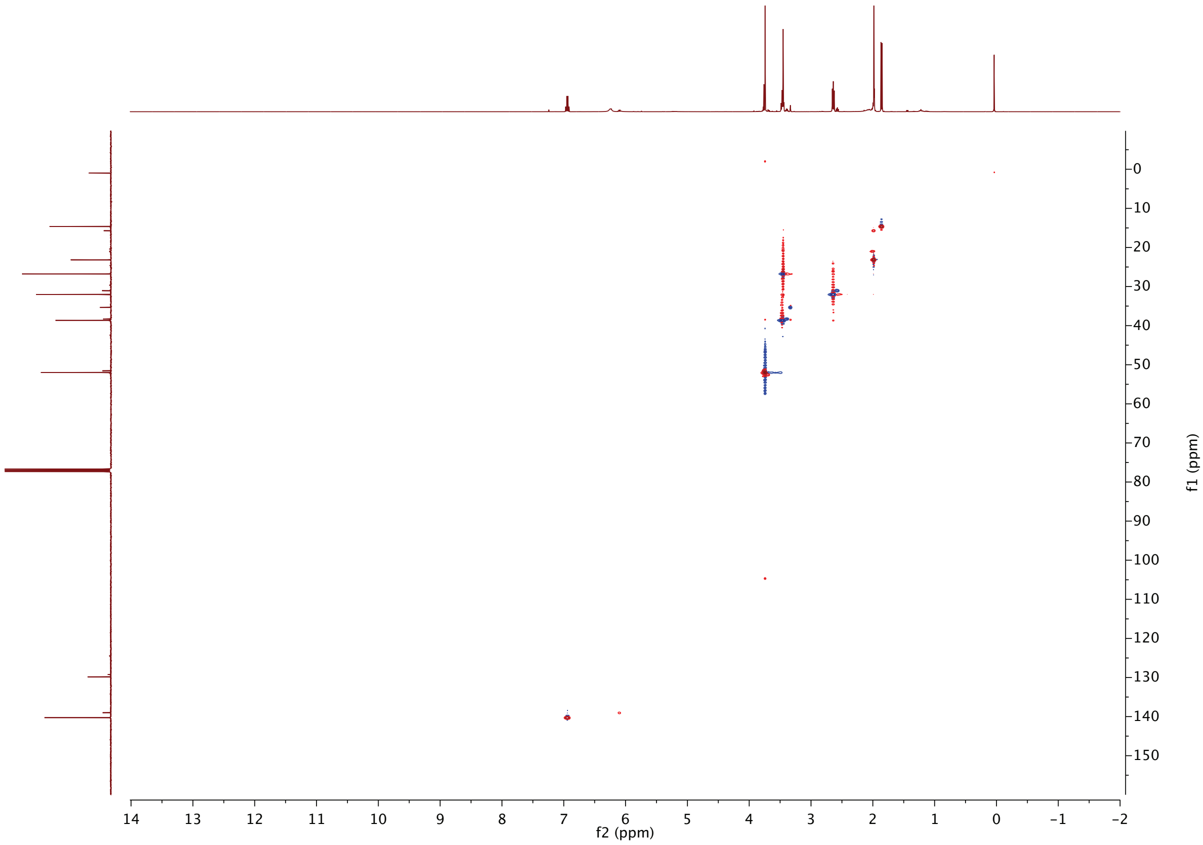

Supplementary Figure 35: gHSQC, compound **2** in $CDCl_3$.

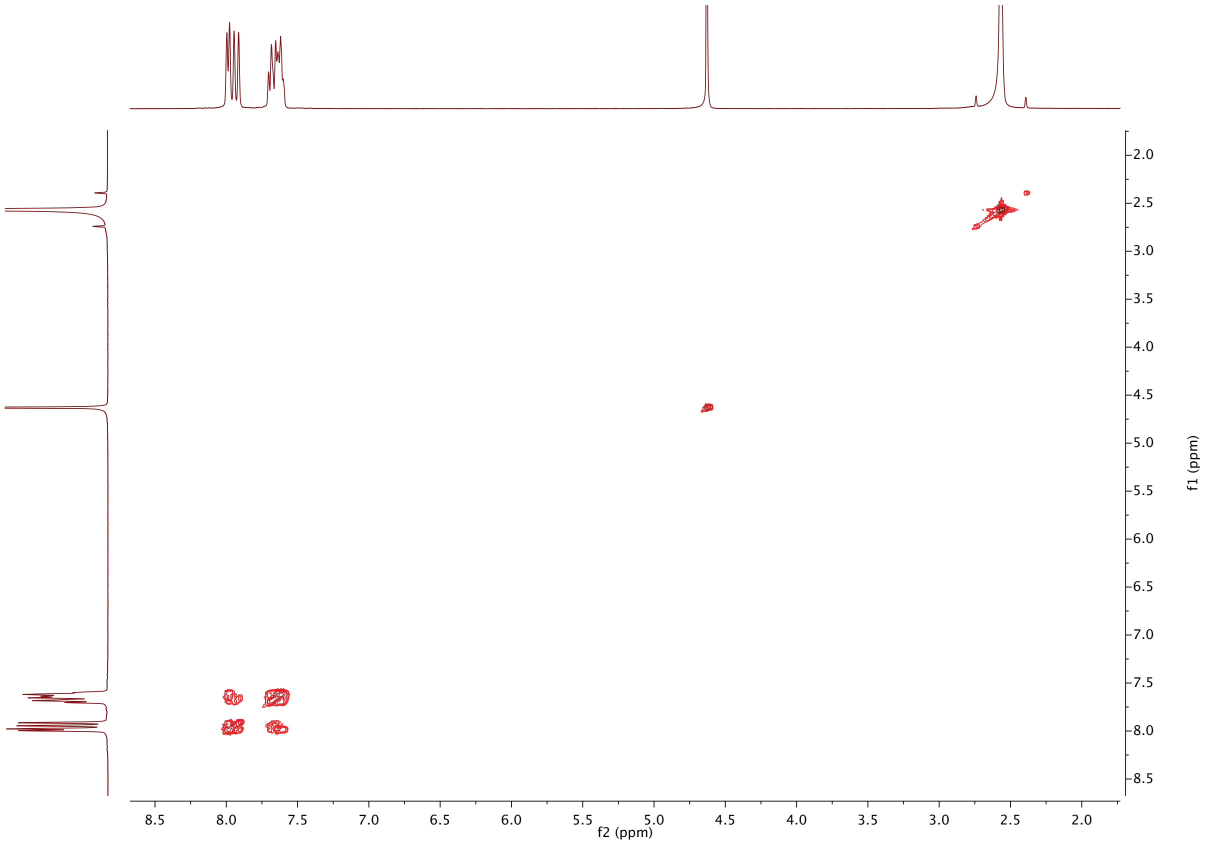

Supplementary Figure 36: gCOSY, compound OTPPTS in D_2O + 5 µL DMSO.

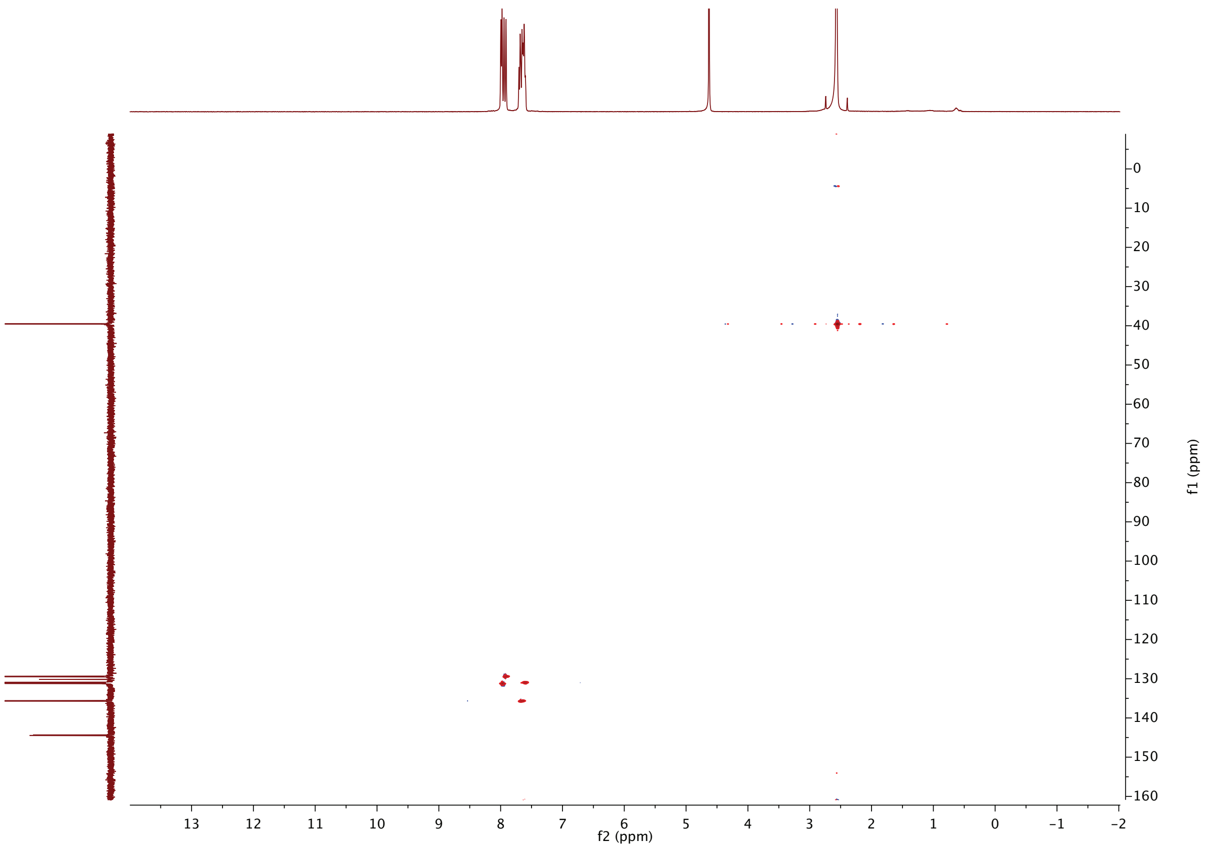

Supplementary Figure 37: gHSQC, compound OTPPTS in D_2O + 5 µL DMSO.

10.0 LC-MS data

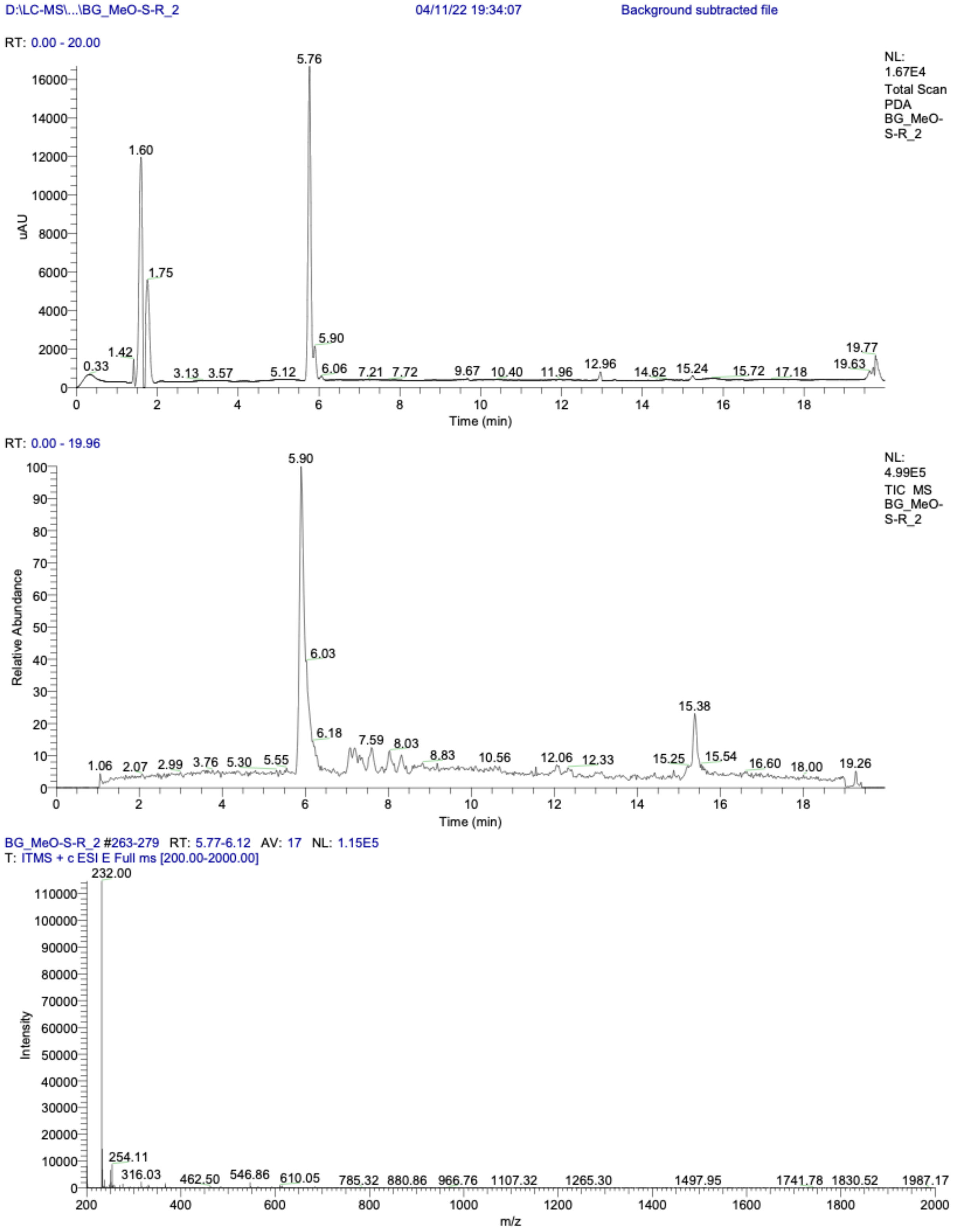

Supplementary Figure 38: LCMS data for compound **2**.

11.0 References

1. Cho, C. & Krische, M. J. Regio-and Stereoselective Construction of γ-Butenolides through Phosphine-Catalyzed Substitution of Morita–Baylis–Hillman Acetates: An Organocatalytic Allylic Alkylation. *Angew. Chemie Int. Ed.* **43**, 6689–6691 (2004).
2. Dmitrenko, O., Thorpe, C. & Bach, R. D. Mechanism of SN2 disulfide bond cleavage by phosphorus nucleophiles. Implications for biochemical disulfide reducing agents. *J. Org. Chem.* **72**, 8298–8307 (2007).
3. Maglangit, F. *et al.* Characterization of the promiscuous N-acyl CoA transferase, LgoC, in legonoxamine biosynthesis. *Org. Biomol. Chem.* **18**, 2219–2222 (2020).
4. Sun, Y. *et al.* Disassemblable micelles based on reduction-degradable amphiphilic graft copolymers for intracellular delivery of doxorubicin. *Biomaterials* **31**, 7124–7131 (2010).
5. Larpent, C. & Patin, H. Nucleophilic addition of water-soluble phosphines on activated olefins. *Tetrahedron* **44**, 6107–6118 (1988).
6. Roy, O., Riahi, A., Hénin, F. & Muzart, J. Synergy or Competition between Palladium-Catalysis and KF/Alumina-Mediation for the Allylic Substitution of the Acetates of Baylis–Hillman Adducts by Phenols. *Tetrahedron* **56**, 8133–8140 (2000).
